# A meta-analysis of pre- and postoperative corticosteroids for reducing the complications following facial reconstructive and aesthetic surgery

**DOI:** 10.1016/j.bjorl.2020.05.015

**Published:** 2020-06-20

**Authors:** Saud A. Aldhabaan, Jibril Y. Hudise, Amani A. Obeid

**Affiliations:** King Saud Univeristy, King Abdulaziz University Hospital, Department of Otolaryngology, Head and Neck Surgery, Riyadh, Saudi Arabia

**Keywords:** Corticosteroid, Edema, Ecchymosis, Facial plastic surgery, Rhinoplasty

## Abstract

**Introduction:**

Edema and ecchymosis after facial plastic surgery are a troublesome concern for both patients and surgeons. Corticosteroid administration is thought to shorten the recovery period and reduce these sequelae. Data regarding the efficacy of corticosteroid administration remains controversial among surgeons.

**Objective:**

We conducted this systematic review and meta-analysis to determine the effect of pre- and postoperative corticosteroids on postoperative complications in patients undergoing facial reconstructive surgery supported with different subgroup analysis.

**Methods:**

A comprehensive literature search of articles was conducted in PubMed, Cochrane Central, SCOPUS, and EBSCO through October 2019. We included all clinical trials in which patients underwent any type of facial plastic surgery to study the effect of corticosteroids on postoperative complications. We performed subgroup analysis according to the types and doses of corticosteroid preparation, in addition to a subgroup analysis of pre- or postoperative corticosteroid usage. All statistical analysis was performed using the RevMan software.

**Results:**

Nineteen studies were included in this systematic review, but only 10 of them were eligible for meta-analysis. The periorbital edema and ecchymosis scores were significantly reduced in the corticosteroids group compared to placebo −0.82, 95% CI (−1.37, −0.26), and -0.95, 95% CI (−1.32, −0.57), respectively. However, these significant differences were not maintained at day 3 and 7. Smaller doses of corticosteroid (8 mg and 10 mg) were associated with smaller differences in the mean score of upper and lower eyelid edema and ecchymosis, while the higher doses were associated with greater differences. Furthermore, preoperative corticosteroid usage significantly reduced the intraoperative bleeding when compared to placebo for higher doses > 50 mg per day (*p* < 0.0001), but not for 8 mg corticosteroid (*p* = 0.06). Adding postoperative steroid dose to the preoperative one was associated with less edema and ecchymosis than preoperative administration alone.

**Conclusion:**

This comprehensive meta-analysis confirms a statistically significant benefit of preoperative corticosteroids. Furthermore, continuing the steroids postoperatively is associated with long-term reduction of complications. Higher doses of corticosteroids are associated with a more significant reduction in edema and ecchymosis, but further studies are recommended to determine the postoperative side effects, including surgical site infection and delayed healing.

## Introduction

Facial plastic surgeries are one of the most commonly performed operative procedures worldwide. Facial surgery includes many types of procedures, including facelifts, rhinoplasty, and maxillofacial plastic surgery.^1^, ^2^, ^3^ Edema, ecchymosis and intraoperative bleeding are the most common postoperative complications after these surgeries.^4^

The severity of edema and ecchymosis is the most important concern as it can delay the healing process of the involved tissues and alter the final intended aesthetic outcome^5^ Various methods and concepts with variable success outcomes were developed to avoid these conditions.^6^

Corticosteroid administration is thought to shorten the recovery period and reduce postoperative edema and ecchymosis.^7^ It provides anti-inflammatory properties by inhibiting the initial process of inflammation that involves the migration of lymphocytes, fibrin deposition, capillary dilatation, and phagocytic activity.^8^ Some studies report that the preoperative administration of steroids is effective, whereas postoperative administration is not.^6^, ^9^

Data regarding the efficacy of corticosteroid administration remains controversial among surgeons, with a debate on the long-term benefit of intraoperative or postoperative corticosteroids administration in patients undergoing facial plastic surgery.^10^, ^11^, ^12^ In most of these studies, various doses and routes of administration were used. Griffies et al. were the first to investigate the effect of corticosteroid on the edema and ecchymosis of facial plastic surgery through a prospective randomized clinical trial.^13^ They reported that a one-time bolus of dexamethasone 10 mg could decrease the facial swelling significantly when compared to the placebo. In 1991, Hoffmann et al. examined the preoperative and postoperative use of dexamethasone 10 mg, and reported it yielded a significant reduction in postoperative eyelid edema, para/intra-nasal edema and ecchymosis.^14^ On the other hand, Berinstein et al.^15^ concluded that the rhinoplasty patients who received dexamethasone experienced increased postoperative edema when compared to controls as assessed by magnetic resonance imaging scans.

Tuncel et al. 2013 reported that pre- and postoperative 10 mg dexamethasone with controlled hypotension considerably reduced postoperative swelling and ecchymosis following rhinoplasty, as well as intraoperative bleeding.^16^ The Turkish study by Gurlek et al. demonstrated that steroids (betamethasone 8 mg, dexamethasone 8 mg, and methylprednisolone 40 mg were not effective in preventing or reducing edema and ecchymosis after open rhinoplasty with osteotomies, with no differences in the levels of ecchymosis or edema among the steroid groups, the tenoxicam group, and the placebo groups being observed.^2^

Recently, in 2018, Sanober et al. showed that preoperative dexamethasone 8 mg could decrease periorbital edema by 50%, compared to placebo 33.3%. By the 7th day postoperatively, only 3.3% in the dexamethasone group had Grade III edema compared to 13.3%in the placebo group.

Considering how common and important facial surgery procedures are, we conducted this systematic review and meta-analysis to resolve the conflicting conclusions reported earlier and to determine the effect of pre- and postoperative corticosteroids on postoperative complications in patients undergoing facial reconstructive surgery.

## Methods

We performed all steps of this systematic review in strict accordance with the Cochrane handbook of systematic reviews and meta-analysis.^17^ We also followed the Preferred reporting items for systematic reviews and meta-analyses (PRISMA statement guidelines) during drafting our manuscript.^18^

### Literature search strategy

We searched the following medical electronic databases: PubMed, Cochrane Central, SCOPUS, and EBSCO through October 2019 using the following query: “(MeSH Facial surgery OR face or facial or nasal dorsum or nose or mouth or ears or lips]” AND “MeSH [Surgery, Plastic] OR [plastic next surgery or craniofacial surgery]” AND “[corticosteroid or glucocorticoid or steroid or dexamethasone or methylprednisolone])”. We also searched the bibliography of eligible studies to find relevant articles.

### Eligibility criteria and study selection

We included all prospective clinical trials (comparative, non-comparative, randomized, or non-randomized) that met all the following criteria: a) Enrolled patients who underwent any type of facial plastic surgery; b) Studies that involved the use of corticosteroids compared or not with another intervention or placebo.

We excluded: a) Observational studies; b) Studies with small sample size (less than ten patients); c) Studies performed on animal models; d) Reviews, case reports, conference abstracts, or case series; and e) Non-English articles and duplicate references.

Eligibility screening was conducted in two steps, each by two independent reviewers (Saud Abdalwahab Aldhabaan and Jibril Yahya Hudise): a) Title and abstract screening for matching the inclusion criteria, and b) Full-text screening for eligibility for meta-analysis. Disagreements were resolved employing the opinion of a third reviewer (Amani Obeid).

### Data extraction

Two independent reviewers (Saud Abdalwahab Aldhabaan and Amani Obeid) extracted the data and another reviewer (Jibril Yahya Hudise) resolved disagreements. The extracted data included the following: a) General characteristics of each study including; study setting, study design, sample size, type of intervention and the doses, type of competitor; b) Patients’ baseline characteristics of each study including: age, gender, race; c) Operation characteristics including: type of surgery, duration of surgery, follow-up period, and postoperative edema assessment; d) Outcomes of interest including swelling (edema), bruising (ecchymosis), intraoperative bleeding and e) Risk of bias criteria.

### Risk of bias assessment

To assess the risk of bias within the included clinical trial, two independent reviewers (Saud Abdalwahab Aldhabaan and Jibril Yahya Hudise) used the Cochrane Risk of Bias (ROB) assessment tool for the randomized clinical trial, clearly described in (chapter 8.5) of the Cochrane handbook of systematic reviews of interventions 5.1.0.^17^, ^18^

The Cochrane risk of bias assessment tool includes the following domain: sequence generation (selection bias), allocation sequence concealment (selection bias), blinding of participants and personnel (performance bias), blinding of outcome assessment (detection bias), incomplete outcome data (attrition bias), selection outcome reporting (reporting bias) and other potential sources of bias. The authors’ judgment is categorized as “low risk”, “high risk” or “unclear risk” of bias.

### Data synthesis

We calculated the Mean Difference (MD) and 95% Confidence Intervals (95% CI), if outcomes were measured in the same way between trials. Standardized Mean Difference (SMD) was calculated in the case of difference in the scoring system.

In some trials, we extracted the data from graphs using PlotDigitizer software.^19^ In the case of missing Standard Deviations (SD), we used the available p-values and the calculator available in the Revman software to determine the SD; if unable to calculate, the highest reported SD from the most similar trial for each outcome was used.

Gurlek et al. 2006^2^ compared three different corticosteroids (betamethasone, dexamethasone and methylprednisolone) with placebo. For such a study, data related to each steroid versus placebo were included in the meta-analysis as a separate entry designated by the name of the study followed by a capital letter (A, B, or C).

We tested for heterogeneity among included studies by the Chi-Square tests and quantified its extent by the I-Square test. In the absence of clinical and statistical heterogeneity (I^2^ less than 50%), the fixed-effect model applied to pool data. In the presence of statistical heterogeneity (I^2^ > 50%), the random-effects model for meta-analysis was applied.

All statistical analysis was performed using the RevMan (version 5.3) for Windows software.

### Subgroup analysis

We performed subgroup analysis acceding to the types and doses of corticosteroid preparation, in addition to a subgroup analysis of per or postoperative corticosteroid.

## Results

### Results of literature search

**Figure 1:**
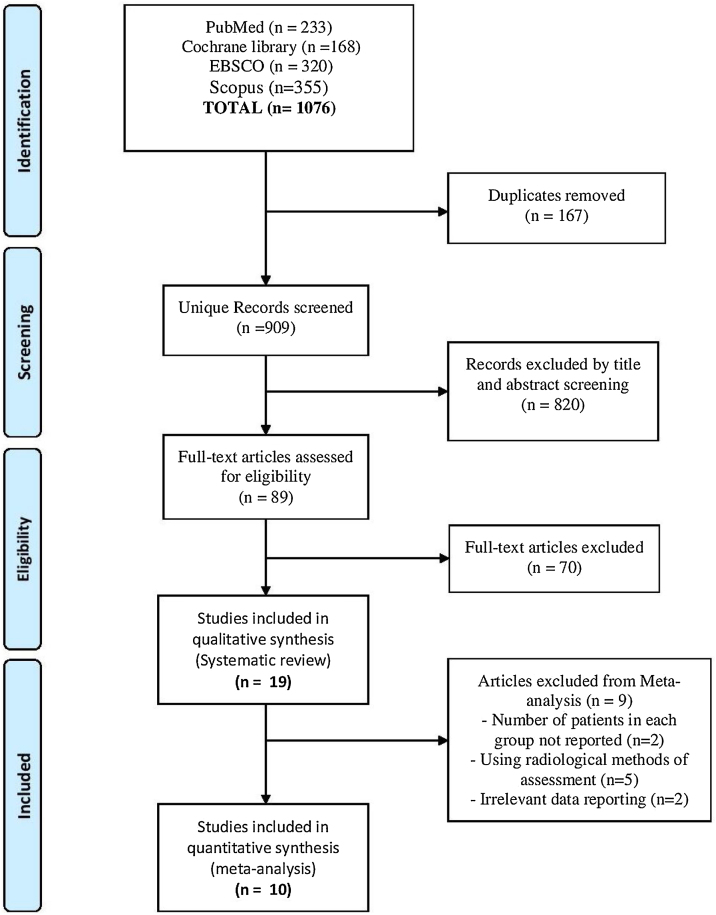
PRISMA flow diagram.

Our search yielded a total of 1076 studies. Following screening and excluding duplicates, 89 studies remained that entered full-text screening. Finally, 19 studies^2^, ^3^, ^4^, ^13^, ^14^, ^15^, ^16^, ^20^, ^21^, ^22^, ^23^, ^24^, ^25^, ^26^, ^27^, ^28^, ^29^, ^30^ were included in this systematic review, but only 10 studies were eligible for the meta-analysis^2^, ^3^, ^4^, ^13^, ^14^, ^16^^,^^20^, ^21^, ^29^, ^30^ as reported in the PRISMA flow diagram (Fig. 1).

### Results of risk of bias assessment

**Figure 2:**
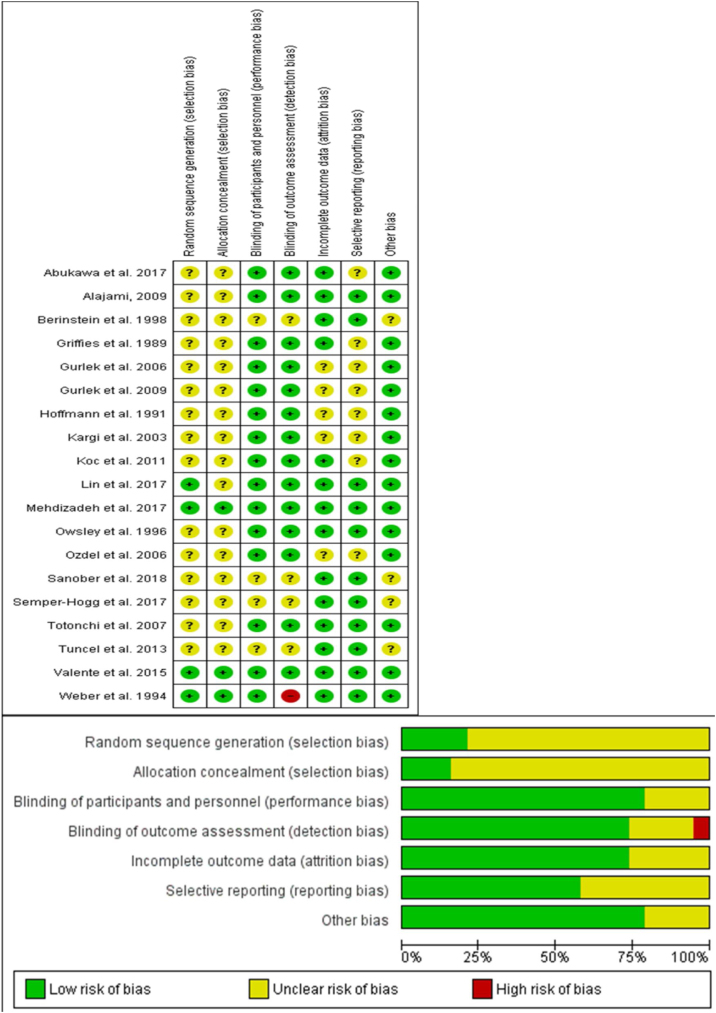
Summary of risk of bias for included trials.

We reported an overall low risk of bias according to ROB assessment tool. A summary of risk of bias assessment was reported in Fig. 2. Most of the included studies did not have any clear information about the random sequence generation and the patients’ allocation. All of the included studies were double-blinded studies except one, which was a triple- blinded study.^30^ All the included studies reported edema and ecchymosis at different days postoperatively.

### Qualitative data analysis

We summarized the baseline characteristics of 19 included studies in Table 1. Of them, 18 studies were prospective randomized controlled studies, and only one study was a retrospective clinical trial.^16^ A total of 843 patients were enrolled in this systematic review with an average of 30–60 patients per study. We identified a significant difference in the type of corticosteroid used regarding the type and dose.

**Table 1:** Baseline characteristics and summary of the included studies as reported in each study.

Study ID		Sitting	Study design		Sample size		Intervention Peri/Intraoperative dose	Postoperative dose	Control		Age, Year		Gender	Type of surgery	Duration of surgery, minutes		Outcome measurement (edema and ecchymosis)	Follow-up visits	Findings
Studies included in the meta-analysis																			
Griffies et al. 1989		USA	Prospective Randomized, double-blind study		30		Immediate preop: Dexamethasone 10 mg IV	No	Placebo (saline)		Median (range), 23 (18–45)		Male 23 (76.7%), Female 7 (23.3%)	Rhinoplasty with osteotomy	NR		Periorbital ecchymosis and edema were evaluated separately using a 4 point graded scale	Day 1	Edema and ecchymosis were significantly reduced in the steroid group.
Gurlek et al. 2006		Turkey	Prospective randomized placebo-controlled double-blind study		40		Immediate preop: betamethasone 8 mg (Group 1), dexamethasone 8 mg (Group 2), methylprednisolone 40 mg (Group 3), tenoxicam 20 mg (Group 4)	Postop: same as peri ×3 days	Placebo (Group 5)		Range; 22‒30		NR	Open rhinoplasty with osteotomies	NR		Digital photographs of each patient were taken and scoring was performed separately for eyelid swelling and ecchymosis by using a graded scale from 0–4.	Days 1, 3, and 7	No differences in the levels of ecchymosis or edema among the steroid groups, the tenoxicam group, and the control groups were observed. Steroids used in these doses were not effective in preventing or reducing edema and ecchymosis after open rhinoplasty with osteotomies.
Gurlek et al. 2009		Turkey	Prospective randomized placebo-controlled double-blind study		40		Immediate preop: single dose methylprednisolone 250 mg (Group 1), single dose methylprednisolone 500 mg (Group 2), 4 doses methylprednisolone 250 mg (Group 3), 4 doses methylprednisolone 5000 mg (Groups 4)	In groups (3 and 4), 250 and 500 mg daily ×3 days	Placebo		Mean 24.5 (range 19–35)		Male 26 (76.7%), Female 14 (23.3%)	Open rhinoplasty with osteotomy	NR		Digital photographs of each patient were taken and scoring was performed separately for eyelid swelling and ecchymosis by using a graded scale from 0 to 4.	Days 1, 3 and 7	Clinically and statistically significant difference was observed regarding ecchymosis and edema, between the placebo and high dose methylprednisolone group.
Hoffmann et al. 1991		USA	Prospective randomized placebo-controlled double-blind study		49		Intraop: Dexamethasone 10 mg IV	Postoper: 5 day taper, 50 mg prednisone by 10 mg daily	Placebo		Range; 15‒70		NR	Closed or Open Rhinoplasty	NR		Upper and lower eyelid edema and periorbital ecchymosis were evaluated using a graded scale from 0-4. Paranasal edema was graded from 0-4 visually and by palpating the soft tissue of the cheek. Intranasal edema was evaluated by anterior rhinoscopic examination and graded from 0‒4 by determining the amount of septal and turbinate edema and the patency of the airway.	1,4,7 days	less postoperative eyelid edema, para/intra-nasal edema and ecchymosis was recorded in patients receiving steroids
Kargi et al. 2003		Turkey	Prospective randomized controlled double-blind study		60		1 h preop: single dose dexamethasone 8 mg IV (Group 1 and 3); Immediate preop: dexamethasone 8 mg IV (Group 2 and 4)	Postop: dexamethasone 8 mg IV 24, 48 h (Group 3 and 4); Immediate postop: dexamethasone 8 mg IV, 24 and 48 h (Group 5)	Nothing (Group 6)		NR		NR	Closed Rhinoplasty	NR		Periorbital ecchymosis and edema were evaluated separately using a 4-point scale	Days 1, 2, 5, 7, 10	Triple-dose administration was found to be more effective in decreasing edema and ecchymosis during the first 5–7 days. Edema and ecchymosis were significantly lower in the steroid groups during the first 2 days compared with the control group. On day 5, edema and ecchymosis were significantly lower in groups 3 and 4 compared with other groups, but there was no difference between them. Group 5 had a significantly higher level of edema and ecchymosis compared with groups 1 through 4 at 24 h and at days 2, 5, and 7.
Koc et al. 2011		Turkey	Prospective randomized controlled trial		40		Preop: single dose of 1 mg/kg IV methylprednisolone (Group I), single dose of 3 mg/kg IV methylprednisolone (Group II)	No	Nothing		NR		Male 22 (55%), Female 18 (45%)	Open Rhinoplasty with Osteotomy	NR		Eyelid edema and periorbital soft-tissue ecchymosis were evaluated separately using a scale of 0–4.	Days 1, 3, and 7	Periorbital edema and ecchymosis were significantly lower in the dexamethasone groups compared with the control group. No significant differences were seen clinically or statistically in preventing or reducing either the periorbital ecchymosis or the periorbital edema between groups I and II.
Mehdizadeh et al. 2017		Iran	Prospective randomized triple-blinded clinical trial		60		Preop: Group D (dexamethasone 8 mg), Group T (tranexamic acid 10 mg/kg), Group DT (dexamethasone 8 mg + tranexamic acid 10 mg/kg)	Postop: three doses every 8 h of corresponding treatments	Nothing (Group P)		Mean 27.35 ± 6		Male 27 (45%), Female 33 (55%)	Primary open rhinoplasty	NR		Photographs were taken after surgery and examined by an independent plastic surgeon for edema and ecchymosis using a scale 0–4	Days 1, 3, and 7	In tranexamic acid, dexamethasone, and tranexamic acid plus dexamethasone groups, edema and ecchymosis were lower; no difference comparing tranexamic acid, dexamethasone, and dexamethasone plus tranexamic acid on any postoperative day; no difference in duration of surgery between any group.
Owsley et al. 1996		USA	Randomized, double-blind study		30		Preop: 500 mg methylprednisolone	Postop: a 6 day oral tapering dose of methyl-prednisone	Nothing		NR		NR	Face lift surgery	NR		Facial swelling was evaluated using a full-face frontal and oblique 35 mm 3 × 5-inch photographic prints taken of every participant preoperatively and on postoperative days 1, 4 (or 6), and 10 on a scale graded 1–4)	Days 1, 4 (or 6), 10	No significant differences in facial swelling between the study group was recorded on any occasion
Ozdel et al. 2006		Turkey	Prospective Randomized, double-blind study		30		Immediate preop: Dexamethasone 10 mg IV	No	Nothing		Study group (23.73 ± 4.58), Control group (25.46 ± 8.70)		Male 15 (50%), Female 15 (50%)	Rhinoplasty with hump removal and bilateral lateral osteotomies	NR		Periorbital edema and ecchymosis were graded, and psychological well-being was measured on a standard visual analog scale. Edema and ecchymosis of the upper and lower eyelids were evaluated separately on a graded scale from 0 to 4 (0 indicates none; 1+, medial; 2+, to pupil; 3+, past pupil; 4+, to lateral canthus)	Days 1, 2	Periorbital edema was significantly reduced on the first 2 postoperative days, and upper eyelid ecchymosis in the dexamethasone group compared with the control. Preoperative steroid administration had no influence on ecchymosis of the lower eyelid
Tuncel et al. 2013		Turkey	Retrospective randomized study		60		Preop: dexamethasone 10 mg/kg IV administered in Group I (single dose), II (2 doses) and III (3 doses).	Postop: (Group II), dexamethasone 10 mg/kg after 24 h; (Group III), same dose dexamethasone immediately before osteotomy and after 24 h	Nothing (Group IV)		Mean 29 (range 23–35)		Male 28 (46.7%), Female 32 (53.3%)	Open rhinoplasty with hump removal and bilateral osteotomies	Group I (87.40 ± 11.65) min, Group II (84.67 ± 10.90) min, Group III (84.40 ± 11.18) min, Group IV (116.67 ± 14.84) min		Degree of eyelid edema and periorbital soft tissue ecchymosis were evaluated separately using a scale of 0−4. Intraoperative blood loss was recorded for each patient. All patients in groups I, II and III were operated under controlled hypotension which was achieved by remifentanil infusion of 0.1‒0.5 microg/kg/min, following a bolus of 1 microg/kg.	Days 1, 2, 5, 7, 10	In the three study groups, eyelid edema and periorbital ecchymosis were significantly decreased at days 7, 10 compared with the control group. There was a statistically significant difference between Group III and other groups at days 5 and 7 regarding lower eyelid edema, upper and lower eyelid ecchymosis. Intraoperative bleeding was more decreased in the study groups compared with the control group.
Studies not included in the meta-analysis																			
Abukawa et al. 2017	Japan			Prospective randomized double-blind controlled study	24	Preop: dexamethasone 8 mg (Group I), 16 mg (Group II)		No		Nothing administered		Control Group (24.0 ± 6.0), Group I (27.3 ± 8.7), Group II (28.6 ± 9.7)	Men 5 (20.8%), Women 19 (79.1%)	Bilateral sagittal split osteotomies (BSSOs)		Control Group (263.1 ± 34.9), Group I (262.0 ± 30.7), Group II (226 ± 30.4)	CT measurements in masseter muscle thickness and buccal soft tissue, maximum incisal opening, sensation of the chin and lower lip region were recorded. Blood examinations, and types of complications were also.	Day 1, 2 and 2 years	In the 16 mg group, rate of increase in the thickness of masseter muscle was lower than the control group. Preoperative levels in the number of lymphocytes were maintained after surgery; whereas there was a reduced number of lymphocytes in the control group.
Alajami, 2009	Kuwait			Prospective randomized placebo-controlled trial	84	Intraop: Dexamethasone 10 mg		Postop: dexamethasone 10 mg given 12 h after surgery		Placebo (5 mL saline)		Study Group (28.1 ± 5.7), Control Group (28.9 ± 6)	Male 28 (33.3%), Female 56 (66.7%)	Open rhinoplasty with hump removal and bilateral lateral osteotomies		NR	Patients were evaluated postoperatively for periorbital edema ± subconjunctival ecchymosis. Absence of edema was graded as 0, edema of lower eyelid alone was graded 1, edema of lower and upper eyelids was graded 2 and edema all around the orbit spreading to face ± subconjunctival ecchymosis was graded 3	Days 1, 2, 5, 7, 10 and 2nd and 3rd week	Preoperative and a 12 h’ postoperative dose of 10 mg of dexamethasone significantly reduced postoperative periorbital edema given during rhinoplasty compared to the control group
Berinstein et al. 1998	USA			Prospective Randomized, double-blind study	20	Preop: dexamethasone 10 mg		No		Nothing		Range; 18‒45	NR	Rhinoplasty		Control Group (Average 135 M in., Study Group (Average 120 Min)	Pre and postoperative magnetic resonance imaging scans were obtained and postoperative edema was quantified as the difference in soft tissue thickness (mm) between the pre and postoperative scans.	‒	On the contrary, the rhinoplasty patients who received dexamethasone had increased postoperative edema when compared to controls.
Semper-Hogg et al. 2017	Germany			Randomized controlled clinical trial	38	Preop: single dose of dexamethasone 40 mg (The patients involved were divided into three subgroups)		No		Nothing		Mean 27.63 (range 16–61)	Male 27 (71.1%), Female 11 (28.9%)	Orthognathic surgery (LeFort I osteotomy (subgroup I), BSSO (subgroup II), bimaxillary osteotomy (subgroup III)		Group I (97.16 ± 41.29), Group II (142.56 ± 29.24), Group II (285 ± 6356)	Postoperative Facial edema was measured using 3D surface scans and neurosensory disturbances were investigated by thermal stimulation.	Days 1, 2, 5, 14 and 90	Facial edema significantly decreased in the study group compared to the control group. The influence of dexamethasone on neurosensory disturbances was not significant for the inferior alveolar nerve or the infraorbital nerve.
Lin et al. 2017	Taiwan			Prospective Randomized controlled, double-blind study	56	Immediate preop: dexamethasone 15 mg IV		No		Immediate preop: dexamethasone 5 mg IV (Group I)		Control Group (20.9 ± 4.8), Study Group (23.2 ± 4.2).	Male 28 (50%), Female 28 (50%)	Orthognathic Surgery		NR	Facial swelling was assessed by 3D images that were recorded at 5 time points: preoperative (T0) and postoperative at 48 ± 6 h (T1), 1 week (T2), 1 month (T3), and 6 months (T4). Facial swelling at T1, T2, and T3 and the reduction in swelling at T2 and T3 compared with that at the baseline (T4) were calculated.	2^nd^ day, 1^st^ week, 1^st^ month, 6^th^ month	The effect of 5 and 15 mg of dexamethasone on facial swelling reduction as well as on nausea and vomiting after orthognathic surgery was not significantly different between study groups.
Sanober et al. 2018	Pakistan			Randomized controlled trial	60	Preop: dexamethasone 8 mg IV (Group I)		Postop: Dexamethasone 8 mg 4 h after surgery		Nothing (Group II)		Mean 26.48 ± 6.07	Male 14 (23.3%), Female 46 (76.6%)	Open Rhinoplasty		NR	Periorbital edema was assessed in terms of on first postoperative day and frontal view pictures were taken and compared on follow up on the 7^th^ day and scores were measured.	Days 1 and 7	Preoperative Dexamethasone decreased periorbital edema by 50% while in control group by 33.3%. The overall by the 7^th^ day control group 13.3% patients had Grade III edema as compared to 3.33% in dexamethasone group.
Totonchi et al. 2007	USA			Randomized controlled trial	48	Intraop: Group P, dexamethasone 10 mg IV; Group A arnica three times a day for 4 days.		Group P: a 6 day oral tapering dose of methyl-prednisone		Nothing (Group C)		Range; 15‒65	Male 11 (22.9%), Female 37 (77.1%)	Primary rhinoplasty with osteotomy		NR	Intensity of ecchymosis, and severity of edema were graded by 3 blinded panelists on days 2 and 8. The panelists rated the extent of ecchymosis on a scale of 0–5, the color density of ecchymosis on a scale of 0–4, and the severity of edema on a scale of 0–3.	Days 2 and 8	Both arnica and corticosteroids reduced edema during the early postoperative period and Arnica did not provide any benefit with regard to extent and intensity of ecchymosis. No significant differences in the ratings of extent and intensity of ecchymosis among the groups on postoperative day 2 but there was a significant difference for the edema rating, with group C demonstrating more swelling compared with groups A and P. On postoperative day 8, group P demonstrated a larger extent and higher intensity of ecchymosis compared with groups A and C and there were no differences in the magnitude of edema by postoperative day 8 among the three groups
Valente et al. 2015	Brazil			Randomized, Double-blind, Placebo-Controlled Clinical Trial	42	Preop: dexamethasone 4 mg/mL IV		No		Preop: 2.5 mL placebo (saline)		Mean 23.12 ± 5.09	Male 3 (7.1%), Female 39 (92.9%)	Rhinoplasty		Mean ± SD (50.17 ± 6.62)	Edema and ecchymosis were assessed using Kara and Gökalan scale by 5 authors. Ecchymosis was assessed according to the affected area as follows: 0 for none, 1 for medium, 2 for to the line of the center of the pupil, 3 for passed the line of the center of the pupil, and 4 for to the lateral corner of the eye. Edema was as follows: 0 for none, 1 for minimum, 2 for to the iris, 3 for reaches the pupil, and 4 for closes the eyes.	Day 7	Dexamethasone significantly reduced the rates of postoperative ecchymosis and edema compared to the controls
Weber et al. 1994	USA			Randomized, Double-blind, Placebo-Controlled Clinical Trial	23	Immediate preop: dexamethasone, 16 mg IV (Group II), dexamethasone, 16 mg IV (Group III)		Postop: 3 IV placebo doses (Group II), 3 doses 8 mg IV dexamethasone (Group III)		Immediate prop: single IV placebo postop: 3 IV placebo doses at 6 h intervals (Group I)		Range; 15‒49	NR	Bilateral Sagittal Split Osteotomies (BSSOs)		186 min (Group I), 164 min (Group II), and 175 min (Group III)	Facial edema was quantified by computer scanning of standardized photographs taken of each patient with a. Five sets of photographic and laboratory data were obtained for each patient: preoperative, day of surgery, and postoperative days 1, 2 and 3. The underlying inflammatory process also was measured using C-reactive protein, erythrocyte sedimentation rate, and complete blood counts.	Days 1, 2, and 3	Dexamethasone administration significantly reduced postoperative edema only on postoperative Day 1 when measured photographically. C-reactive protein was significantly reduced on postoperative days 1,2 and 3 in both dexamethasone groups and no significant difference was found between the two dexamethasone groups.

NR, not reported.

Dexamethasone 8 mg was administered in five studies, while dexamethasone 10 mg was administered in seven studies. Lin et al. 2017^25^ and Semper-Hogg et al. 2017^24^ reported the effect of a higher dexamethasone dose of 15 mg and 40 mg, respectively. A lower dexamethasone dose of 4 mg was investigated by Valente et al. 2015.^27^ Gurlek et al. 2006^2^ was the only study that reported the effect of betamethasone 8 mg for reducing the edema and ecchymosis in rhinoplasty. They compared equivalent doses of three different corticosteroids (dexamethasone 8 mg, betamethasone 8 mg, and methylprednisolone 40 mg) with a placebo. In 2009, Gurlek and colleagues^3^ investigated the use of a higher dose of corticosteroids (methylprednisolone 250 mg, 500 mg, and 5000 mg).

Four studies investigated the methylprednisolone with different doses. Eleven studies investigated the efficacy of combined pre- and postoperative corticosteroid, while eight studies reported preoperative corticosteroid only. Fifteen studies reported the results after rhinoplastic surgery, two studies after orthognathic surgery,^24^, ^25^ one study after bilateral sagittal split osteotomies,^22^ and one studiy after facelift surgery.^20^ The summary of the findings of each included study was reported in the same Table 1.

### Quantitative data analysis

A total of 439 patients from 10 clinical trials were included in the meta-analysis. All of these studies reported the assessment of upper and lower eyelid ecchymosis and edema postoperatively using the four-point scale. The most commonly used corticosteroid doses were 8 mg and 10 mg. The higher doses were pooled in one group (corticosteroid > 50 mg). We pooled the results of postoperative ecchymosis and edema at day 1, 3, 5, 7 and 10. The detailed results of subgroup analysis according to the types and doses of the used corticosteroid as well as a subgroup analysis of pre and postoperative corticosteroids are presented in Supplementary Tables 1 and 2.

### Upper eyelid edema

**Figure 3:**
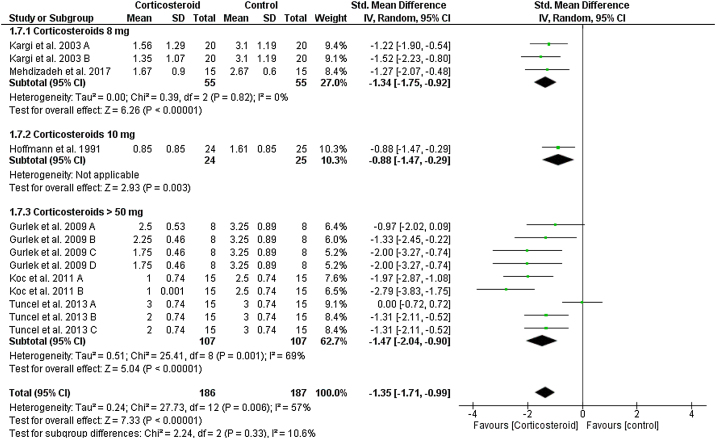
Forest plot of upper eyelid edema on Day 1.

On the first postoperative day, the overall SMD favored corticosteroids over placebo in terms of upper eyelid edema total score (SMD = -1.35, 95% CI: -1.71, -0.99). Tests for subgroup differences between corticosteroids dose 8 mg, 10 mg, and > 50 mg were not significant, Chi² = 2.24, *p*  = 0.33, I² = 10.6%. Pooled studies were homogenous for dose 8 mg (I^2^ = 0%), but heterogonous for dose > 50 mg (I^2^ = 69%). Heterogeneity was best resolved by exclusion of Tuncel et al. 2013 A (single dose of 10 mg/kg group), I² = 22% (Fig. 3).

At day 3 postoperatively, corticosteroid was continuously reduced the score of upper eyelid edema compared to placebo (SMD = -1.42, 95% CI: -1.82, -1.02) with no heterogeneity (I² = 0%), except for dose 8 mg subgroup (I² = 87%) (Supplementary Fig. 1). The same significant results were obtained on day 7 postoperatively (Supplementary Fig. 2). Tuncel et al. 2013 reported a significant difference between corticosteroid and placebo at day 10 postoperatively.

### Lower eyelid edema

On the first postoperative day, the overall SMD favored corticosteroids over placebo in terms of lower eyelid edema total score (SMD = -1.14, 95% CI: -1.36, -0.91). Test for subgroup differences between corticosteroids dose 8 mg, 10 mg, and > 50 mg was significant, Chi² = 6.73, *p*  = 0.03, I² = 70.3%.

**Figure 4:**
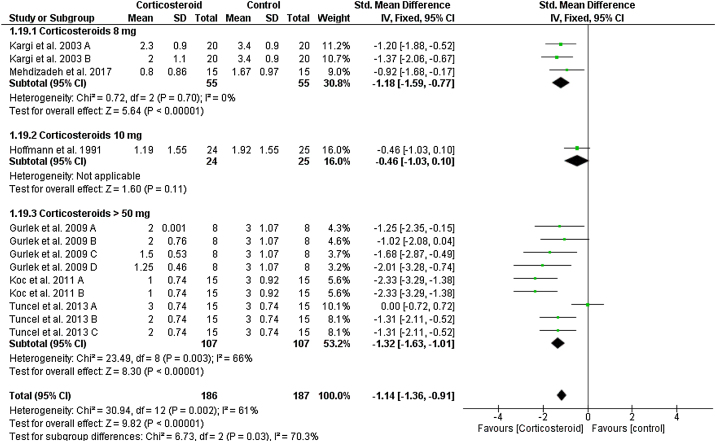
Forest plot of lower eyelid edema on Day 1.

Pooled studies were homogenous for dose 8 mg (I^2^ = 0%), but heterogonous for dose > 50 mg (I^2^ = 66%). Heterogeneity was best resolved by exclusion of Tuncel et al. 2013 A (single dose of 10 mg/kg group), I² = 6% (Fig. 4). We obtained the same significance at day 3; the overall SMD favored corticosteroids 8 mg (SMD = -0.97, 95% CI: -1.74, -0.21) and > 50 mg (SMD = -1.26, 95% CI: -1.66, -0.86). Tests for subgroup differences was not significant: Chi² = 0.42, *p*  = 0.52, I² = 0% (Supplementary Fig. 3). This significant difference was maintained to day 5, 7 and 10 postoperatively as seen in Supplementary Figures 4‒6.

### Upper eyelid ecchymosis

**Figure 5:**
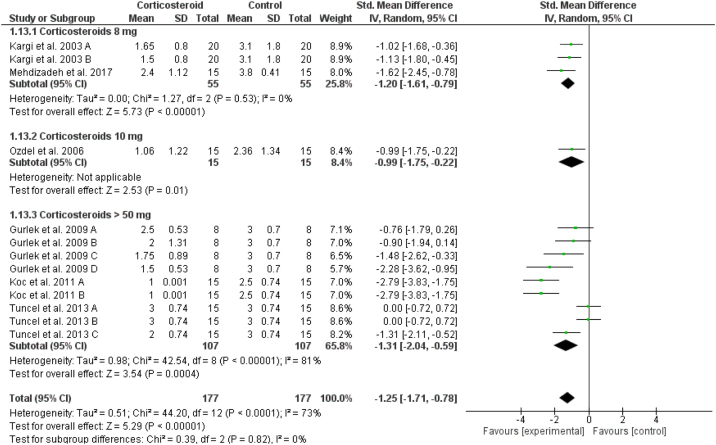
Forest plot of upper eyelid ecchymosis on Day 1.

In comparison to the placebo, corticosteroids reduced the mean score of upper eyelid ecchymosis significantly at day 1 postoperatively (overall SMD = -1.25, 95% CI: -1.71, -0.78). Test for subgroup differences between corticosteroids dose 8 mg, 10 mg, and > 50 mg was not significant, Chi² = 0.39, *p*  = 0.82, I² = 0% (Fig. 5). The same significant results were revealed for postoperative Days 3, 5, 7 and 10 for the overall effect estimate as presented in Supplementary Figures 7‒10. Tuncel et al. 2013 reported a non-significant (*p* =  0.32) difference between corticosteroid (10 mg/kg) and placebo at day 5 postoperatively.

### Lower eyelid ecchymosis

**Figure 6:**
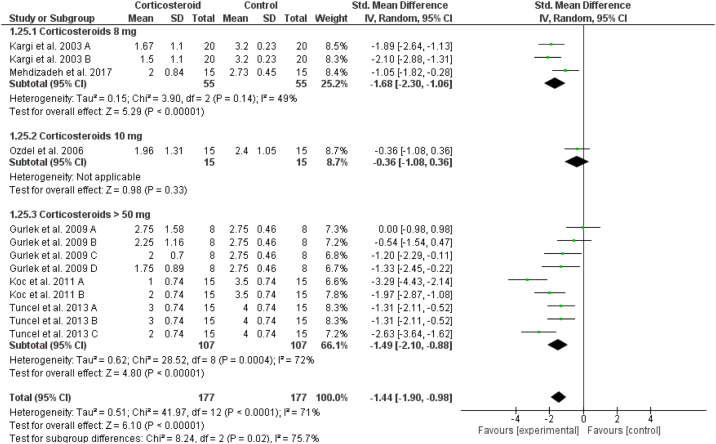
Forest plot of lower eyelid ecchymosis on Day 1.

The total score of lower eyelid ecchymosis decreased significantly in the corticosteroids group in comparison to placebo at day 1 (overall SMD = -1.44, 95% CI: -1.90, -0.98). Tests for subgroup differences between corticosteroids dose 8 mg, 10 mg and > 50 mg were significant, Chi² = 8.24, *p* =  0.02, I² = 75.7% (Fig. 6). The same significant results were revealed for postoperative days 3, 5, 7 and 10 for the overall effect estimate as presented in Supplementary Figures 11‒14.

### Unspecified edema

**Figure 7:**
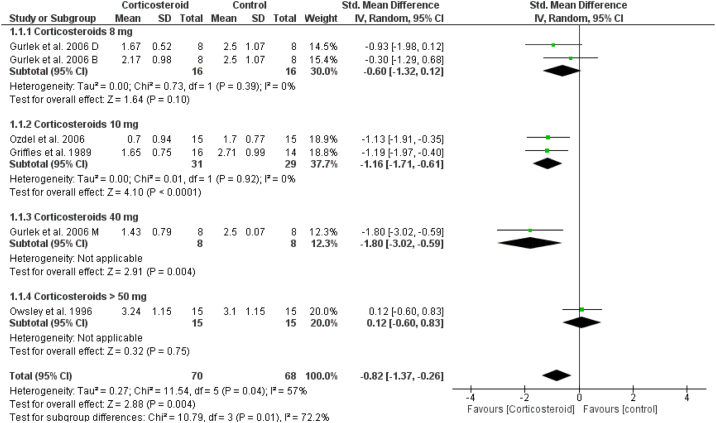
Forest plot of unspecified edema.

On the first day, the overall effect size of the edema score revealed that edema was significantly reduced in corticosteroids group compared to placebo (SMD = -0.82, 95% CI: -1.37, -0.26). This was not achieved in corticosteroid 8 mg group, but achieved in higher doses as presented in Fig. 7. This significance was not maintained at day 3 and 7 (Supplementary Figs. 15‒16).

### Unspecified ecchymosis

**Figure 8:**
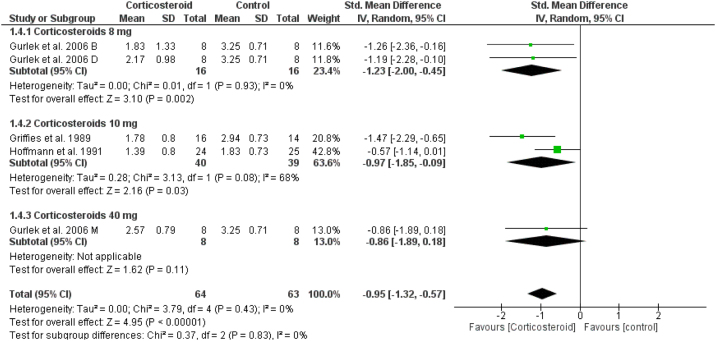
Forest plot of unspecified ecchymosis.

In comparison to the placebo, the corticosteroids group reduced the postoperative ecchymosis at day one (SMD = -0.95, 95% CI: -1.32, -0.57) (Fig. 8). While on days 3 and 7, the results were not significant (Supplementary Figs. 17‒18).

### Intraoperative bleeding

**Figure 9:**
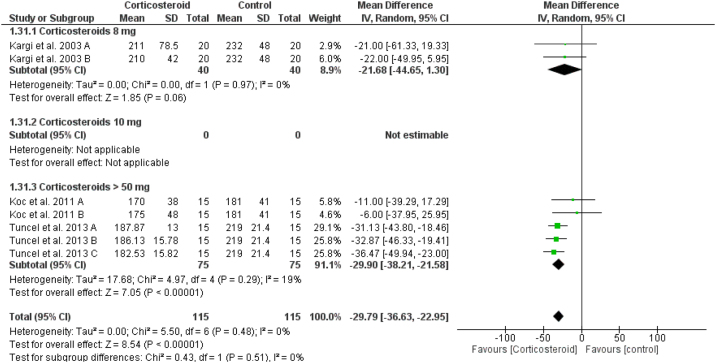
Forest plot of intraoperative bleeding.

Preoperative corticosteroid significantly reduced the intraoperative bleeding when compared to placebo (overall SMD = -29.79 mL, 95% CI: -36.63, -22.95) without difference or heterogeneity between the corticosteroid doses (test for subgroup differences: Chi² = 0.53, *p* =  0.47, I² = 0%) (Fig. 9).

## Discussion

The present systematic review included 19 clinical trials. Of them, only 10 studies were included in the quantitative evidence synthesis. Our meta-analysis provides Class 1 evidence that corticosteroid has a beneficial effect in reducing postoperative edema and ecchymosis. In addition, preoperative corticosteroids decreased intraoperative bleeding. Although postoperative edema and ecchymosis have commonly occurred after facial plastic surgery in which steroids are commonly used, small numbers of studies were conducted to establish its effectiveness and determine the preferred dose and type to be administered.

On the first postoperative day, smaller doses of corticosteroid (8 mg and 10 mg) were associated with smaller differences in the mean score of upper and lower eyelid edema and ecchymosis, while the higher doses were associated with greater differences. None of the included studies reported any adverse events related to the higher or lower doses of corticosteroid.

Furthermore, preoperative corticosteroid administration significantly reduced the intraoperative bleeding when compared to placebo for higher doses > 50 mg per day (*p* <  0.0001), but not for 8mg corticosteroid (*p* = 0.06). Adding postoperative steroid dose to the preoperative one was associated with a decrease in edema and ecchymosis than when given preoperatively alone. Using higher doses of methylprednisolone could improve the postoperative edema and ecchymosis efficiently at day one postoperatively rather than any other steroid. Using a higher methylprednisolone dose was not associated with any intraoperative or postoperative adverse events, but it significantly decreased the intraoperative bleeding.^3^, ^4^

None of the included studies reported the results of postoperative pain, healing duration, patient satisfaction, or the quality of life. Corticosteroid has a significant effect in reducing pain following facial plastic, dentoalveolar, and maxillofacial surgeries.^31^

In the present meta-analysis, the included studies used the same scale of 0–4 for evaluating the degree of edema and ecchymosis with minimal differences in the description of each grade; therefore, we used the standardized mean difference for each comparison.

Five studies were excluded from the meta-analysis due to their use of different radiological methods, but they reported important results. Abukawa et al. 2017^22^ used the CT measurements in masseter muscle thickness and buccal soft tissue to assess the postoperative edema and revealed that the rate of increase in the masseter muscle thickness in the corticosteroid group was significantly lower than that in the control group. On the other hand, Berinstein et al. 1998^15^ concluded that the rhinoplasty patients who received dexamethasone had increased postoperative edema when compared to controls as assessed by magnetic resonance imaging scans that quantified the difference in soft tissue thickness between the pre- and postoperative scans.

Using 3D surface scans, Semper-Hogg et al. 2017^24^ showed that facial edema significantly decreased postoperatively in the dexamethasone 40 mg group compared to the control group, while lower doses of dexamethasone (5 and 15 mg) did not reduce the facial swelling after orthognathic surgery. Weber and colleagues^28^ quantified the facial edema by computer scanning of five sets of standardized photographs and revealed that dexamethasone (8 and 16 mg) administration significantly reduced the postoperative edema.

Alajami and colleagues^23^ reported a different method of assessment: the absence of edema was graded as 0, edema of lower eyelid alone was Graded 1, edema of lower and upper eyelids was Graded 2 and edema all around the orbit spreading to the face ± subconjunctival ecchymosis was Graded 3. The result showed that there was highly significant difference in the presence of different grades of edema in the corticosteroid group in all assessment days (*p* <  0.001). Grade 1 edema was present in the corticosteroid group at day 1 (43.2%), day 2 (29.5%), day 5 (13.6%) and was completely absent from 7th postoperative day onward. In the placebo group, on the 7th postoperative day, 33.3% of the patients still had Grade 1 and 16.7% had Grade 2 edema. Moreover, 16.7% in the placebo group continued to show Grade 1 edema on the 10th postoperative day.

Regarding the intensity of ecchymosis and severity of edema, Totonchi et al. 2007^26^ showed that dexamethasone 10 mg reduced edema during the early postoperative period with no significant differences in the ratings of extent and intensity of ecchymosis on postoperative day 2, but there was a significant difference for the edema rating, with the control group demonstrating more swelling compared with dexamethasone group. On postoperative day 8, the dexamethasone group demonstrated a larger extent and higher intensity of ecchymosis compared with the control group and there were no differences in the magnitude of edema by postoperative Day 8.

Tuncel et al. 2013^16^ reported that dexamethasone with controlled hypotension considerably reduced intraoperative bleeding, postoperative swelling and ecchymosis of rhinoplasty. Including this study in our meta-analysis was associated with significant heterogeneity because it reported the comparative data as median and interquartile range, which required farther data transformation to be pooled with other studies. Removing Tunnel’s study from the analysis led to resolving the heterogeneity and was not associated with the change in the overall significance in all outcomes.

The overall quality of the evidence of the included studies is high. The main concern is the small sample size of each article. According to the American Society of Plastic Surgeons report 2018, Nose reshaping is one of 2018′s top 5 cosmetic surgical procedures (213,000 surgeries in 2018).^32^

Although we are presenting the first large comprehensive systematic review (19 included studies) to investigate the effect of corticosteroids on reducing the post-facial plastic surgery complications, we reported a consistent result with the previous Cochran review in 2014^33^ and with other systematic reviews that investigated the use of steroids in rhinoplasty.^34^, ^35^ The Cochran review reported that a single preoperative dose of 10 mg dexamethasone decreased edema and ecchymosis over the first two postoperative days; this difference was not observed after this period, while high doses of methylprednisolone decreased both ecchymosis and edema at days 1, 3 and 7 postoperatively.

Most of the included studies were heterogeneous regarding the numbers and dosage of pre-and postoperative corticosteroids as well as the type of the steroids used.. Studies by Abukawa et al.,^22^ Griffies et al.,^13^ Koc et al.,^4^ and Ozdel et al.^21^ described giving steroid preoperatively while studies by Tuncel et al.,^16^ Mehdizadeh et al.,^30^ Kargi et al.,^29^ Hoffmann et al.,^14^ and Alajami,^23^ gave steroids as both a pre- and postoperative dose. Therefore, we performed subgroup analysis to pool the similar groups together in one meta-analysis model and provide an overall effect estimate as seen in supplementary Tables 1‒2. Most of the subgroups were underpowered and the result cannot be generalized due to the small numbers of included studies. We recommend conducting well-designed randomized controlled trials with a large sample size to investigate the effect of timing (pre and postoperative) and dosage (single and multiple) and type of corticosteroid given for facial plastic surgery.

In conclusion, this comprehensive meta-analysis confirms a statistically significant benefit of the preoperative administration of corticosteroid when compared to placebo. Furthermore, continuing the steroids postoperatively is associated with long-term reduction of complications, including edema, ecchymosis, and intraoperative bleeding. Finally, higher doses of corticosteroids are associated with a more significant reduction in edema and ecchymosis, but further studies are recommended to determine the postoperative side effects such as surgical site infection and delayed healing.

## Conflicts of interest

The authors declare no conflicts of interest.
