# Supplementary material for: A meta-analysis of pre- and postoperative corticosteroids for reducing the complications following facial reconstructive and aesthetic surgery
Source: Braz J Otorhinolaryngol. 2020 Jun 20;88(1):63–82. doi: 10.1016/j.bjorl.2020.05.015 (PMC9422590; doi:10.1016/j.bjorl.2020.05.015)

BJORL-D-20-00117 – Supplementary material

**Supplementary Table 1** Summary of the effect estimate with subgrouping according the corticosteroid used versus placebo regarding the oedema and ecchymosis at day 1, 3, 5, and 7.

| **Type and dose of corticosteroid** | **Study ID** | **Std mean diff** | **SE** | **95% CI** | | **p-value** |
| --- | --- | --- | --- | --- | --- | --- |
|  |  |  |  | **Lower CI** | **Upper CI** |  |
| Oedema upper eyelid at day 1 | |  |  |  |  |  |
| Dexamethasone 10 mg | Hoffmann et al. 1991 | -2.983 | -0.894 | 0.090 | -1.482 | 0.003 |
| Dexamethasone 10 mg/kg | Tuncel et al. 2013 A | 0.000 | 0.000 | 0.133 | -0.716 | 1.000 |
|  | Tuncel et al. 2013 B | -3.339 | -1.351 | 0.164 | -2.145 | 0.001 |
|  | Tuncel et al. 2013 C | -1.351 | -1.351 | -2.145 | -0.558 | 0.001 |
|  | Overall Effect Size | -1.351 | -0.858 | -2.145 | -0.558 | 0.001 |
| Dexamethasone 8 mg | Kargi et al. 2003 A | -3.594 | -1.241 | 0.119 | -1.918 | 0.000 |
|  | Kargi et al. 2003 B | -4.291 | -1.546 | 0.130 | -2.253 | 0.000 |
|  | Mehdizadeh et al. 2017 | -3.250 | -1.307 | 0.162 | -2.096 | 0.001 |
|  | Overall Effect Size | -6.441 | -1.365 | 0.045 | -1.780 | 0.000 |
| Methylprednisolone 1 mg/kg | Koc et al. 2011 A | -4.512 | -2.027 | 0.202 | -2.908 | 0.000 |
| Methylprednisolone 250 mg | Gurlek et al. 2009 A | -1.926 | -1.024 | 0.283 | -2.066 | 0.054 |
|  | Gurlek et al. 2009 C | -3.390 | -2.117 | 0.390 | -3.342 | 0.001 |
|  | Overall Effect Size | -3.664 | -1.483 | 0.164 | -2.277 | 0.000 |
| Methylprednisolone 3 mg/kg | Koc et al. 2011 B | -5.514 | -2.867 | 0.270 | -3.886 | 0.000 |
| Methylprednisolone 500 mg | Gurlek et al. 2009 B | -2.526 | -1.412 | 0.312 | -2.507 | 0.012 |
|  | Gurlek et al. 2009 D | -3.390 | -2.117 | 0.390 | -3.342 | 0.001 |
|  | Overall Effect Size | -4.143 | -1.725 | 0.173 | -2.542 | 0.000 |
| Oedema upper eyelid at day 3 | |  |  |  |  |  |
| Dexamethasone 8 mg | Mehdizadeh et al. 2017 | -3.927 | -1.663 | 0.179 | -2.494 | 0.000 |
| Methylprednisolone 1 mg/kg | Koc et al. 2011 A | -3.339 | -1.351 | 0.164 | -2.145 | 0.001 |
| Methylprednisolone 250 mg | Gurlek et al. 2009 A | -2.413 | -1.334 | 0.306 | -2.418 | 0.016 |
|  | Gurlek et al. 2009 C | -2.666 | -1.511 | 0.321 | -2.622 | 0.008 |
|  | Overall Effect Size | -3.589 | -1.421 | 0.157 | -2.196 | 0.000 |
| Methylprednisolone 500 mg | Gurlek et al. 2009 B | -2.666 | -1.511 | 0.321 | -2.622 | 0.008 |
|  | Gurlek et al. 2009 D | -2.666 | -1.511 | 0.321 | -2.622 | 0.008 |
|  | Overall Effect Size | -3.770 | -1.511 | 0.161 | -2.297 | 0.000 |
| Oedema upper eyelid at day 5 | |  |  |  |  |  |
| Dexamethasone 8mg | Mehdizadeh et al. 2017 | -3.927 | -1.663 | 0.179 | -2.494 | 0.000 |
| Methylprednisolone 1 mg/kg | Koc et al. 2011 A | -3.339 | -1.351 | 0.164 | -2.145 | 0.001 |
| Methylprednisolone 250 mg | Gurlek et al. 2009 A | -2.413 | -1.334 | 0.306 | -2.418 | 0.016 |
|  | Gurlek et al. 2009 C | -2.666 | -1.511 | 0.321 | -2.622 | 0.008 |
|  | Overall Effect Size | -3.589 | -1.421 | 0.157 | -2.196 | 0.000 |
| Methylprednisolone 500 mg | Gurlek et al. 2009 B | -2.666 | -1.511 | 0.321 | -2.622 | 0.008 |
|  | Gurlek et al. 2009 D | -2.666 | -1.511 | 0.321 | -2.622 | 0.008 |
|  | Overall Effect Size | -3.770 | -1.511 | 0.161 | -2.297 | 0.000 |
| Oedema upper eyelid at day 7 | |  |  |  |  |  |
| Dexamethasone 10 mg | Hoffmann et al. 1991 | -5.034 | -1.671 | 0.110 | -2.321 | 0.000 |
| Dexamethasone 10 mg/kg | Tuncel et al. 2013 A | 0.000 | 0.000 | 0.133 | -0.716 | 1.000 |
|  | Tuncel et al. 2013 B | 0.000 | 0.000 | 0.133 | -0.716 | 1.000 |
|  | Tuncel et al. 2013 C | 0.000 | 0.000 | 0.133 | -0.716 | 1.000 |
|  | Overall Effect Size | -2.699 | -0.480 | 0.032 | -0.829 | 0.007 |
| Dexamethasone 8 mg | Kargi et al. 2003 A | -3.351 | -1.143 | 0.116 | -1.811 | 0.001 |
|  | Kargi et al. 2003 B | -6.384 | -2.883 | 0.204 | -3.767 | 0.000 |
|  | Mehdizadeh et al. 2017 | -3.481 | -1.423 | 0.167 | -2.224 | 0.000 |
|  | Overall Effect Size | -7.358 | -1.667 | 0.051 | -2.111 | 0.000 |
| Methylprednisolone 250 mg | Gurlek et al. 2009 A | -1.457 | -0.754 | 0.268 | -1.768 | 0.145 |
|  | Gurlek et al. 2009 C | -3.447 | -2.174 | 0.398 | -3.410 | 0.001 |
|  | Overall Effect Size | -3.313 | -1.325 | 0.160 | -2.109 | 0.001 |
| Methylprednisolone 500 mg | Gurlek et al. 2009 B | -2.200 | -1.194 | 0.295 | -2.258 | 0.028 |
|  | Gurlek et al. 2009 D | -3.762 | -2.519 | 0.448 | -3.831 | 0.000 |
|  | Overall Effect Size | -4.078 | -1.719 | 0.178 | -2.546 | 0.000 |
| Ecchymosis upper eyelid at day 1 | |  |  |  |  |  |
| Dexamethasone 10 mg | Ozdel et al. 2006 | -2.615 | -1.015 | 0.150 | -1.775 | 0.009 |
| Dexamethasone 10 mg/kg | Tuncel et al. 2013 A | 0.000 | 0.000 | 0.133 | -0.716 | 1.000 |
|  | Tuncel et al. 2013 B | 0.000 | 0.000 | 0.133 | -0.716 | 1.000 |
|  | Tuncel et al. 2013 C | -3.339 | -1.351 | 0.164 | -2.145 | 0.001 |
|  | Overall Effect Size | -2.846 | -0.540 | 0.036 | -0.912 | 0.004 |
| Dexamethasone 8 mg | Kargi et al. 2003 A | -3.089 | -1.041 | 0.114 | -1.701 | 0.002 |
|  | Kargi et al. 2003 B | -3.366 | -1.149 | 0.116 | -1.818 | 0.001 |
|  | Mehdizadeh et al. 2017 | -3.921 | -1.660 | 0.179 | -2.490 | 0.000 |
|  | Overall Effect Size | -5.903 | -1.232 | 0.044 | -1.641 | 0.000 |
| Methylprednisolone 1 mg/kg | Koc et al. 2011 A | -5.514 | -2.867 | 0.270 | -3.886 | 0.000 |
| Methylprednisolone 250 mg | Gurlek et al. 2009 A | -1.549 | -0.805 | 0.270 | -1.824 | 0.121 |
|  | Gurlek et al. 2009 C | -2.734 | -1.561 | 0.326 | -2.681 | 0.006 |
|  | Overall Effect Size | -2.986 | -1.148 | 0.148 | -1.901 | 0.003 |
| Methylprednisolone 3 mg/kg | Koc et al. 2011 B | -5.514 | -2.867 | 0.270 | -3.886 | 0.000 |
| Methylprednisolone 500mg | Gurlek et al. 2009 B | -1.805 | -0.952 | 0.278 | -1.986 | 0.071 |
|  | Gurlek et al. 2009 D | -3.674 | -2.416 | 0.432 | -3.705 | 0.000 |
|  | Overall Effect Size | -3.707 | -1.525 | 0.169 | -2.332 | 0.000 |
| Ecchymosis upper eyelid at day 3 | |  |  |  |  |  |
| Dexamethasone 8 mg | Mehdizadeh et al. 2017 | -3.797 | -1.590 | 0.175 | -2.412 | 0.000 |
| Methylprednisolone 1 mg/kg | Koc et al. 2011 A | -3.021 | -1.198 | 0.157 | -1.975 | 0.003 |
| Methylprednisolone 250 mg | Gurlek et al. 2009 A | -1.726 | -0.906 | 0.276 | -1.935 | 0.084 |
|  | Gurlek et al. 2009 C | -3.244 | -1.980 | 0.373 | -3.176 | 0.001 |
|  | Overall Effect Size | -3.424 | -1.363 | 0.158 | -2.143 | 0.001 |
| Methylprednisolone 3 mg/kg | Koc et al. 2011 B | -5.006 | -2.396 | 0.229 | -3.333 | 0.000 |
| Methylprednisolone 500 mg | Gurlek et al. 2009 B | -2.077 | -1.116 | 0.289 | -2.170 | 0.038 |
|  | Gurlek et al. 2009 D | -3.390 | -2.117 | 0.390 | -3.342 | 0.001 |
|  | Overall Effect Size | -3.785 | -1.542 | 0.166 | -2.341 | 0.000 |
| Ecchymosis upper eyelid at day 5 | |  |  |  |  |  |
| Dexamethasone 10 mg/kg | Tuncel et al. 2013 A | 0.000 | 0.000 | 0.133 | -0.716 | 1.000 |
|  | Tuncel et al. 2013 B | 0.000 | 0.000 | 0.133 | -0.716 | 1.000 |
|  | Tuncel et al. 2013 C | -3.339 | -1.351 | 0.164 | -2.145 | 0.001 |
|  | Overall Effect Size | -1.796 | -0.391 | 0.047 | -0.818 | 0.072 |
| Dexamethasone 8 mg | Kargi et al. 2003 A | -4.020 | -1.423 | 0.125 | -2.117 | 0.000 |
|  | Kargi et al. 2003 B | -5.512 | -2.214 | 0.161 | -3.001 | 0.000 |
|  | Overall Effect Size | -6.661 | -1.769 | 0.071 | -2.289 | 0.000 |
| Ecchymosis upper eyelid at day 7 | |  |  |  |  |  |
| Dexamethasone 10 mg/kg | Tuncel et al. 2013 A | 0.000 | 0.000 | 0.133 | -0.716 | 1.000 |
|  | Tuncel et al. 2013 B | 0.000 | 0.000 | 0.133 | -0.716 | 1.000 |
|  | Tuncel et al. 2013 C | -2.889 | -1.137 | 0.155 | -1.908 | 0.004 |
|  | Overall Effect Size | -1.585 | -0.342 | 0.047 | -0.765 | 0.113 |
| Dexamethasone 8 mg | Kargi et al. 2003 A | -3.202 | -1.084 | 0.115 | -1.748 | 0.001 |
|  | Kargi et al. 2003 B | -5.375 | -2.127 | 0.157 | -2.902 | 0.000 |
|  | Mehdizadeh et al. 2017 | -4.465 | -1.995 | 0.200 | -2.871 | 0.000 |
|  | Overall Effect Size | -7.365 | -1.642 | 0.050 | -2.079 | 0.000 |
| Methylprednisolone 1 mg/kg | Koc et al. 2011 A | -3.021 | -1.198 | 0.157 | -1.975 | 0.003 |
| Methylprednisolone 250 mg | Gurlek et al. 2009 A | -2.029 | -1.087 | 0.287 | -2.137 | 0.042 |
|  | Gurlek et al. 2009 C | -3.762 | -2.519 | 0.448 | -3.831 | 0.000 |
|  | Overall Effect Size | -3.935 | -1.646 | 0.175 | -2.466 | 0.000 |
| Methylprednisolone 3 mg/kg | Koc et al. 2011 B | -3.021 | -1.198 | 0.157 | -1.975 | 0.003 |
| Methylprednisolone 500 mg | Gurlek et al. 2009 B | -2.878 | -1.672 | 0.337 | -2.810 | 0.004 |
|  | Gurlek et al. 2009 D | -3.762 | -2.519 | 0.448 | -3.831 | 0.000 |
|  | Overall Effect Size | -4.639 | -2.035 | 0.192 | -2.895 | 0.000 |
| Oedema Lower eyelid at day 1 | |  |  |  |  |  |
| Dexamethasone 10 mg/kg | Hoffmann et al. 1991 | -1.626 | -0.471 | 0.084 | -1.039 | 0.104 |
| Dexamethasone 10 mg | Tuncel et al. 2013 A | 0.000 | 0.000 | 0.133 | -0.716 | 1.000 |
|  | Tuncel et al. 2013 B | -3.339 | -1.351 | 0.164 | -2.145 | 0.001 |
|  | Tuncel et al. 2013 C | -3.339 | -1.351 | 0.164 | -2.145 | 0.001 |
|  | Overall Effect Size | -3.932 | -0.699 | 0.032 | -1.048 | 0.000 |
| Dexamethasone 8 mg | Kargi et al. 2003 A | -3.548 | -1.222 | 0.119 | -1.897 | 0.000 |
|  | Kargi et al. 2003 B | -3.952 | -1.393 | 0.124 | -2.084 | 0.000 |
|  | Mehdizadeh et al. 2017 | -2.464 | -0.949 | 0.148 | -1.704 | 0.014 |
|  | Overall Effect Size | -5.792 | -1.202 | 0.043 | -1.609 | 0.000 |
| Methylprednisolone 1 mg/kg | Koc et al. 2011 A | -5.006 | -2.396 | 0.229 | -3.333 | 0.000 |
| Methylprednisolone 250 mg | Gurlek et al. 2009 A | -2.395 | -1.322 | 0.305 | -2.403 | 0.017 |
|  | Gurlek et al. 2009 C | -3.009 | -1.777 | 0.349 | -2.934 | 0.003 |
|  | Overall Effect Size | -3.804 | -1.534 | 0.163 | -2.324 | 0.000 |
| Methylprednisolone 3 mg/kg | Koc et al. 2011 B | -5.006 | -2.396 | 0.229 | -3.333 | 0.000 |
| Methylprednisolone 500 mg | Gurlek et al. 2009 B | -2.014 | -1.078 | 0.286 | -2.126 | 0.044 |
|  | Gurlek et al. 2009 D | -3.398 | -2.125 | 0.391 | -3.351 | 0.001 |
|  | Overall Effect Size | -3.739 | -1.520 | 0.165 | -2.317 | 0.000 |
| Oedema Lower eyelid at day 3 | |  |  |  |  |  |
| Dexamethasone 8 mg | Mehdizadeh et al. 2017 | -2.583 | -1.000 | 0.150 | -1.759 | 0.010 |
| Methylprednisolone 1 mg/kg | Koc et al. 2011 A | -4.337 | -1.911 | 0.194 | -2.775 | 0.000 |
| Methylprednisolone 250 mg | Gurlek et al. 2009 A | -2.029 | -1.087 | 0.287 | -2.137 | 0.042 |
|  | Gurlek et al. 2009 C | -2.690 | -1.529 | 0.323 | -2.643 | 0.007 |
|  | Overall Effect Size | -3.322 | -1.295 | 0.152 | -2.059 | 0.001 |
| Methylprednisolone 3 mg/kg | Koc et al. 2011 B | -4.337 | -1.911 | 0.194 | -2.775 | 0.000 |
| Methylprednisolone 500 mg | Gurlek et al. 2009 B | -2.029 | -1.087 | 0.287 | -2.137 | 0.042 |
|  | Gurlek et al. 2009 D | 0.000 | 0.000 | 0.250 | -0.980 | 1.000 |
|  | Overall Effect Size | -1.385 | -0.506 | 0.134 | -1.222 | 0.166 |
| Oedema Lower eyelid at day 5 | |  |  |  |  |  |
| Dexamethasone 10 mg/kg | Tuncel et al. 2013 A | 0.000 | 0.000 | 0.133 | -0.716 | 1.000 |
|  | Tuncel et al. 2013 B | -4.336 | -1.911 | 0.194 | -2.775 | 0.000 |
|  | Tuncel et al. 2013 C | -5.477 | -2.828 | 0.267 | -3.840 | 0.000 |
|  | Overall Effect Size | -5.049 | -1.247 | 0.061 | -1.731 | 0.000 |
| Dexamethasone 8 mg | Kargi et al. 2003 A | -2.014 | -0.654 | 0.105 | -1.290 | 0.044 |
|  | Kargi et al. 2003 B | -4.822 | -1.811 | 0.141 | -2.547 | 0.000 |
|  | Overall Effect Size | -4.677 | -1.149 | 0.060 | -1.630 | 0.000 |
| Methylprednisolone 250mg | Gurlek et al. 2009 A | -2.413 | -1.334 | 0.306 | -2.418 | 0.016 |
|  | Gurlek et al. 2009 C | -3.139 | -1.887 | 0.361 | -3.065 | 0.002 |
|  | Overall Effect Size | -3.901 | -1.587 | 0.166 | -2.385 | 0.000 |
| Methylprednisolone 500 mg | Gurlek et al. 2009 B | -2.666 | -1.511 | 0.321 | -2.622 | 0.008 |
|  | Gurlek et al. 2009 D | -3.762 | -2.519 | 0.448 | -3.831 | 0.000 |
|  | Overall Effect Size | -4.466 | -1.932 | 0.187 | -2.780 | 0.000 |
| Oedema Lower eyelid at day 7 | |  |  |  |  |  |
| Dexamethasone 10 mg | Hoffmann et al. 1991 | -0.123 | -0.035 | 0.082 | -0.595 | 0.902 |
| Dexamethasone 10 mg/kg | Tuncel et al. 2013 A | 0.000 | 0.000 | 0.133 | -0.716 | 1.000 |
|  | Tuncel et al. 2013 B | 0.000 | 0.000 | 0.133 | -0.716 | 1.000 |
|  | Tuncel et al. 2013 C | -2.889 | -1.137 | 0.155 | -1.908 | 0.004 |
|  | Overall Effect Size | -1.339 | -0.231 | 0.030 | -0.568 | 0.181 |
| Dexamethasone 8 mg | Kargi et al. 2003 A | -1.482 | -0.475 | 0.103 | -1.104 | 0.138 |
|  | Kargi et al. 2003 B | -3.643 | -1.261 | 0.120 | -1.940 | 0.000 |
|  | Mehdizadeh et al. 2017 | -2.983 | -1.180 | 0.157 | -1.956 | 0.003 |
|  | Overall Effect Size | -4.587 | -0.927 | 0.041 | -1.324 | 0.000 |
| Methylprednisolone 1 mg/kg | Koc et al. 2011 A | -3.339 | -1.351 | 0.164 | -2.145 | 0.001 |
| Methylprednisolone 250 mg | Gurlek et al. 2009 A | -2.413 | -1.334 | 0.306 | -2.418 | 0.016 |
|  | Gurlek et al. 2009 C | -3.139 | -1.887 | 0.361 | -3.065 | 0.002 |
|  | Overall Effect Size | -3.901 | -1.587 | 0.166 | -2.385 | 0.000 |
| Methylprednisolone 3 mg/kg | Koc et al. 2011 B | -3.339 | -1.351 | 0.164 | -2.145 | 0.001 |
| Methylprednisolone 500 mg | Gurlek et al. 2009 B | -2.666 | -1.511 | 0.321 | -2.622 | 0.008 |
|  | Gurlek et al. 2009 D | -3.762 | -2.519 | 0.448 | -3.831 | 0.000 |
|  | Overall Effect Size | -4.466 | -1.932 | 0.187 | -2.780 | 0.000 |
| Ecchymosis Lower eyelid at day 1 | |  |  |  |  |  |
| Dexamethasone 10 mg | Ozdel et al. 2006 | -1.006 | -0.371 | 0.136 | -1.092 | 0.314 |
| Dexamethasone 10 mg/kg | Tuncel et al. 2013 A | -3.339 | -1.351 | 0.164 | -2.145 | 0.001 |
|  | Tuncel et al. 2013 B | -3.339 | -1.351 | 0.164 | -2.145 | 0.001 |
|  | Tuncel et al. 2013 C | -5.351 | -2.703 | 0.255 | -3.693 | 0.000 |
|  | Overall Effect Size | -6.153 | -1.269 | 0.043 | -1.673 | 0.000 |
| Dexamethasone 8 mg | Kargi et al. 2003 A | -5.033 | -1.925 | 0.146 | -2.675 | 0.000 |
|  | Kargi et al. 2003 B | -5.396 | -2.139 | 0.157 | -2.916 | 0.000 |
|  | Mehdizadeh et al. 2017 | -2.771 | -1.083 | 0.153 | -1.850 | 0.006 |
|  | Overall Effect Size | -7.620 | -1.715 | 0.051 | -2.156 | 0.000 |
| Methylprednisolone 1 mg/kg | Koc et al. 2011 A | -5.939 | -3.378 | 0.324 | -4.493 | 0.000 |
| Methylprednisolone 250 mg | Gurlek et al. 2009 A | 0.000 | 0.000 | 0.250 | -0.980 | 1.000 |
|  | Gurlek et al. 2009 C | -2.311 | -1.266 | 0.300 | -2.340 | 0.021 |
|  | Overall Effect Size | -1.558 | -0.575 | 0.136 | -1.299 | 0.119 |
| Methylprednisolone 3 mg/kg | Koc et al. 2011 B | -4.512 | -2.027 | 0.202 | -2.908 | 0.000 |
| Methylprednisolone 500 mg | Gurlek et al. 2009 B | -1.111 | -0.567 | 0.260 | -1.566 | 0.266 |
|  | Gurlek et al. 2009 D | -2.526 | -1.412 | 0.312 | -2.507 | 0.012 |
|  | Overall Effect Size | -2.524 | -0.951 | 0.142 | -1.689 | 0.012 |
| Ecchymosis Lower eyelid at day 3 | |  |  |  |  |  |
| Dexamethasone 8 mg | Mehdizadeh et al. 2017 | -2.519 | -0.973 | 0.149 | -1.730 | 0.012 |
| Methylprednisolone 1 mg/kg | Koc et al. 2011 A | -5.700 | -3.074 | 0.291 | -4.131 | 0.000 |
| Methylprednisolone 250 mg | Gurlek et al. 2009 A | -1.484 | -0.769 | 0.268 | -1.785 | 0.138 |
|  | Gurlek et al. 2009 C | -1.765 | -0.929 | 0.277 | -1.961 | 0.078 |
|  | Overall Effect Size | -2.296 | -0.848 | 0.136 | -1.572 | 0.022 |
| Methylprednisolone 3 mg/kg | Koc et al. 2011 B | -5.006 | -2.396 | 0.229 | -3.333 | 0.000 |
| Methylprednisolone 500 mg | Gurlek et al. 2009 B | -2.200 | -1.194 | 0.295 | -2.258 | 0.028 |
|  | Gurlek et al. 2009 D | -3.447 | -2.174 | 0.398 | -3.410 | 0.001 |
|  | Overall Effect Size | -3.916 | -1.611 | 0.169 | -2.417 | 0.000 |
| Ecchymosis Lower eyelid at day 5 | |  |  |  |  |  |
| Dexamethasone 10 mg/kg | Tuncel et al. 2013 A | 0.000 | 0.000 | 0.133 | -0.716 | 1.000 |
|  | Tuncel et al. 2013 B | -2.219 | -0.846 | 0.145 | -1.592 | 0.027 |
|  | Overall Effect Size | -1.535 | -0.405 | 0.070 | -0.921 | 0.125 |
| Dexamethasone 8 mg | Kargi et al. 2003 A | -2.623 | -0.868 | 0.109 | -1.516 | 0.009 |
|  | Kargi et al. 2003 B | -4.080 | -1.450 | 0.126 | -2.146 | 0.000 |
|  | Mehdizadeh et al. 2017 | 0.000 | 0.000 | 0.133 | -0.716 | 1.000 |
|  | Overall Effect Size | -3.917 | -0.790 | 0.041 | -1.186 | 0.000 |
| Ecchymosis Lower eyelid at day 7 | |  |  |  |  |  |
| Dexamethasone 10 mg/kg | Tuncel et al. 2013 A | 0.000 | 0.000 | 0.133 | -0.716 | 1.000 |
|  | Tuncel et al. 2013 B | 0.000 | 0.000 | 0.133 | -0.716 | 1.000 |
|  | Tuncel et al. 2013 C | -3.339 | -1.351 | 0.164 | -2.145 | 0.001 |
|  | Overall Effect Size | -1.796 | -0.391 | 0.047 | -0.818 | 0.072 |
| Dexamethasone 8 mg | Kargi et al. 2003 A | -2.154 | -0.702 | 0.106 | -1.340 | 0.031 |
|  | Kargi et al. 2003 B | -4.159 | -1.486 | 0.128 | -2.186 | 0.000 |
|  | Mehdizadeh et al. 2017 | -3.958 | -1.681 | 0.180 | -2.514 | 0.000 |
|  | Overall Effect Size | -5.774 | -1.209 | 0.044 | -1.620 | 0.000 |
| Methylprednisolone 1 mg/kg | Koc et al. 2011 A | -3.339 | -1.351 | 0.164 | -2.145 | 0.001 |
| Methylprednisolone 250 mg | Gurlek et al. 2009 A | -1.486 | -0.770 | 0.269 | -1.786 | 0.137 |
|  | Gurlek et al. 2009 C | -3.139 | -1.887 | 0.361 | -3.065 | 0.002 |
|  | Overall Effect Size | -3.175 | -1.246 | 0.154 | -2.015 | 0.001 |
| Methylprednisolone 3 mg/kg | Koc et al. 2011 B | -3.339 | -1.351 | 0.164 | -2.145 | 0.001 |
| Methylprednisolone 500 mg | Gurlek et al. 2009 B | -1.926 | -1.024 | 0.283 | -2.066 | 0.054 |
|  | Gurlek et al. 2009 D | -2.974 | -1.748 | 0.345 | -2.900 | 0.003 |
|  | Overall Effect Size | -3.423 | -1.350 | 0.155 | -2.123 | 0.001 |

**Supp. Table 2** Summary of the effect estimate with subgrouping according the post-operative corticosteroid dose used vs. placebo regarding the oedema and ecchymosis at Day 1, 3, 5 and 7.

| **Post-operative**  **corticosteroid dose** | **Study ID** | | | **Std Mean Diff** | **SE** | **95% CI** | | **p-value** |
| --- | --- | --- | --- | --- | --- | --- | --- | --- |
|  |  |  |  |  |  | **Lower CI** | **Upper CI** |  |
| Oedema upper eyelid at day 1 |  | | | | | | | |
| Dexamethasone 10 mg/kg, No | Tuncel et al. 2013 A | | | 0.00 | 0.37 | -0.72 | 0.72 | 1.00 |
| Dexamethasone 10 mg, Single Dose | Hoffmann et al. 1991 | | | -0.89 | 0.30 | -1.48 | -0.31 | 0.00 |
| Dexamethasone 10 mg/kg, Single Dose | Tuncel et al. 2013 B | | | -1.35 | 0.40 | -2.14 | -0.56 | 0.00 |
|  | Tuncel et al. 2013 C | | | 0.00 | 0.37 | -0.72 | 0.72 | 1.00 |
|  | Overall effect size | | | -0.74 | 0.20 | -1.13 | -0.34 | 0.00 |
| Dexamethasone 8mg, Multiple (2 Doses) | Kargi et al. 2003 B | | | -1.55 | 0.36 | -2.25 | -0.84 | 0.00 |
| Dexamethasone 8mg, Multiple (3 Doses) | Mehdizadeh et al. 2017 | | | -1.31 | 0.40 | -2.10 | -0.52 | 0.00 |
| Dexamethasone 8mg, No | Kargi et al. 2003 A | | | -1.24 | 0.35 | -1.92 | -0.56 | 0.00 |
| Methylprednisolone 1mg/kg, No | Koc et al. 2011 A | | | -2.03 | 0.45 | -2.91 | -1.15 | 0.00 |
| Methylprednisolone 250mg, Multiple (3 Doses) | Gurlek et al. 2009 C | | | -2.12 | 0.62 | -3.34 | -0.89 | 0.00 |
| Methylprednisolone 250mg, No | Gurlek et al. 2009 A | | | -1.02 | 0.53 | -2.07 | 0.02 | 0.05 |
| Methylprednisolone 3mg/kg, No | Koc et al. 2011 B | | | -2.87 | 0.52 | -3.89 | -1.85 | 0.00 |
| Methylprednisolone 500mg, Multiple (3 Doses) | Gurlek et al. 2009 D | | | -2.12 | 0.62 | -3.34 | -0.89 | 0.00 |
| Methylprednisolone 500mg, No | Gurlek et al. 2009 B | | | -1.41 | 0.56 | -2.51 | -0.32 | 0.01 |
| Oedema upper eyelid at day 3 |  | | | | | | | |
| Dexamethasone 8mg, Multiple (3 Doses) | Mehdizadeh et al. 2017 | | | -1.66 | 0.42 | -2.49 | -0.83 | 0.00 |
| Methylprednisolone 1mg/kg, No | Koc et al. 2011 A | | | -1.35 | 0.40 | -2.14 | -0.56 | 0.00 |
| Methylprednisolone 250mg, Multiple (3 Doses) | Gurlek et al. 2009 C | | | -1.51 | 0.57 | -2.62 | -0.40 | 0.01 |
| Methylprednisolone 250mg, No | Gurlek et al. 2009 A | | | -1.33 | 0.55 | -2.42 | -0.25 | 0.02 |
| Methylprednisolone 500 mg, Multiple (3 Doses) | Gurlek et al. 2009 D | | | -1.51 | 0.57 | -2.62 | -0.40 | 0.01 |
| Methylprednisolone 500 mg, No | Gurlek et al. 2009 B | | | -1.51 | 0.57 | -2.62 | -0.40 | 0.01 |
| Oedema upper eyelid at day 5 |  | | | | | | |  |
| Dexamethasone mg/kg, Single Dose | Tuncel et al. 2013 B | | | -1.35 | 0.40 | -2.14 | -0.56 | 0.00 |
|  | Tuncel et al. 2013 C | | | -1.91 | 0.44 | -2.77 | -1.05 | 0.00 |
|  | Overall effect size | | | -1.61 | 0.30 | -2.19 | -1.02 | 0.00 |
| Dexamethasone 8 mg, Multiple (2 Doses) | Kargi et al. 2003 B | | | -2.57 | 0.43 | -3.41 | -1.73 | 0.00 |
| Dexamethasone 8 mg, No | Kargi et al. 2003 A | | | -1.00 | 0.34 | -1.66 | -0.34 | 0.00 |
| Dexamethasone, No | Tuncel et al. 2013 A | | | -1.35 | 0.40 | -2.14 | -0.56 | 0.00 |
| Oedema upper eyelid at day 7 |  | | | | | | | |
| Dexamethasone 10 mg, No | Tuncel et al. 2013 A | | | 0.00 | 0.37 | -0.72 | 0.72 | 1.00 |
| Dexamethasone 10 mg, Single Dose | Hoffmann et al. 1991 | | | -1.67 | 0.33 | -2.32 | -1.02 | 0.00 |
| Dexamethasone 10 mg/kg, single dose | Tuncel et al. 2013 B | | | 0.00 | 0.37 | -0.72 | 0.72 | 1.00 |
|  | Tuncel et al. 2013 C | | | 0.00 | 0.37 | -0.72 | 0.72 | 1.00 |
|  | Overall effect size | | | -0.63 | 0.20 | -1.03 | -0.23 | 0.00 |
| Dexamethasone 8 mg, multiple (2 Doses) | Kargi et al. 2003 B | | | -2.88 | 0.45 | -3.77 | -2.00 | 0.00 |
| Dexamethasone 8mg, multiple (3 Doses) | Mehdizadeh et al. 2017 | | | -1.42 | 0.41 | -2.22 | -0.62 | 0.00 |
| Dexamethasone 8 mg, No | Kargi et al. 2003 A | | | -1.14 | 0.34 | -1.81 | -0.47 | 0.00 |
| Methylprednisolone 250 mg, multiple (3 Doses) | Gurlek et al. 2009 C | | | -2.17 | 0.63 | -3.41 | -0.94 | 0.00 |
| Methylprednisolone 250mg, No | Gurlek et al. 2009 A | | | -0.75 | 0.52 | -1.77 | 0.26 | 0.15 |
| Methylprednisolone 500mg, Multiple (3 Doses) | Gurlek et al. 2009 D | | | -2.52 | 0.67 | -3.83 | -1.21 | 0.00 |
| Methylprednisolone 500 mg, No | Gurlek et al. 2009 B | | | -1.19 | 0.54 | -2.26 | -0.13 | 0.03 |
| Ecchymosis upper eyelid at day 1 | | |  | | | | | |
| Dexamethasone 10 mg, No | Ozdel et al. 2006 | | | -1.01 | 0.39 | -1.77 | -0.25 | 0.01 |
| Dexamethasone 10 mg/kg, No | Tuncel et al. 2013 A | | | 0.00 | 0.37 | -0.72 | 0.72 | 1.00 |
|  | Overall effect size | | | -0.48 | 0.27 | -1.00 | 0.04 | 0.07 |
| Dexamethasone 10 mg/kg, single dose | Tuncel et al. 2013 B | | | 0.00 | 0.37 | -0.72 | 0.72 | 1.00 |
|  | Tuncel et al. 2013 C | | | -1.35 | 0.40 | -2.14 | -0.56 | 0.00 |
|  | Overall effect size | | | -0.61 | 0.27 | -1.14 | -0.08 | 0.03 |
| Dexamethasone 8 mg, multiple (2 Doses) | Kargi et al. 2003 B | | | -1.15 | 0.34 | -1.82 | -0.48 | 0.00 |
| Dexamethasone 8 mg, multiple (3 Doses) | Mehdizadeh et al. 2017 | | | -1.66 | 0.42 | -2.49 | -0.83 | 0.00 |
| Dexamethasone 8 mg, No | Kargi et al. 2003 A | | | -1.04 | 0.34 | -1.70 | -0.38 | 0.00 |
| Methylprednisolone 1mg/kg, No | Koc et al. 2011 A | | | -2.87 | 0.52 | -3.89 | -1.85 | 0.00 |
| Methylprednisolone 250 mg, multiple (3 Doses) | Gurlek et al. 2009 C | | | -1.56 | 0.57 | -2.68 | -0.44 | 0.01 |
| Methylprednisolone 250 mg, No | Gurlek et al. 2009 A | | | -0.81 | 0.52 | -1.82 | 0.21 | 0.12 |
| Methylprednisolone 3mg/kg, No | Koc et al. 2011 B | | | -2.87 | 0.52 | -3.89 | -1.85 | 0.00 |
| Methylprednisolone 500 mg, Multiple (3 Doses) | Gurlek et al. 2009 D | | | -2.42 | 0.66 | -3.70 | -1.13 | 0.00 |
| Methylprednisolone 500 mg, No | Gurlek et al. 2009 B | | | -0.95 | 0.53 | -1.99 | 0.08 | 0.07 |
| Ecchymosis upper eyelid at day 3 | |  | | | | | | |
| Dexamethasone 8 mg, multiple (3 Doses) | Mehdizadeh et al. 2017 | | | -1.59 | 0.42 | -2.41 | -0.77 | 0.00 |
| Methylprednisolone 1mg/kg, No | Koc et al. 2011 A | | | -1.20 | 0.40 | -1.98 | -0.42 | 0.00 |
| Methylprednisolone 250 mg, multiple (3 Doses) | Gurlek et al. 2009 C | | | -1.98 | 0.61 | -3.18 | -0.78 | 0.00 |
| Methylprednisolone 250 mg, No | Gurlek et al. 2009 A | | | -0.91 | 0.53 | -1.94 | 0.12 | 0.08 |
| Methylprednisolone 3mg/kg, No | Koc et al. 2011 B | | | -2.40 | 0.48 | -3.33 | -1.46 | 0.00 |
| Methylprednisolone 500 mg, multiple (3 Doses) | Gurlek et al. 2009 D | | | -2.12 | 0.62 | -3.34 | -0.89 | 0.00 |
| Methylprednisolone 500mg, No | Gurlek et al. 2009 B | | | -1.12 | 0.54 | -2.17 | -0.06 | 0.04 |
| cchymosis upper eyelid at day 5 |  | | | | | | | |
| Dexamethasone 10 mg/kg, No | Tuncel et al. 2013 A | | | 0.00 | 0.37 | -0.72 | 0.72 | 1.00 |
| Dexamethasone 10 mg/kg, single dose | Tuncel et al. 2013 B | | | 0.00 | 0.37 | -0.72 | 0.72 | 1.00 |
|  | Tuncel et al. 2013 C | | | -1.35 | 0.40 | -2.14 | -0.56 | 0.00 |
|  | Overall effect size | | | -0.61 | 0.27 | -1.14 | -0.08 | 0.03 |
| Dexamethasone 8 mg, multiple (2 Doses) | Kargi et al. 2003 B | | | -2.21 | 0.40 | -3.00 | -1.43 | 0.00 |
| Dexamethasone 8 mg, No | Kargi et al. 2003 A | | | -1.42 | 0.35 | -2.12 | -0.73 | 0.00 |
| Ecchymosis upper eyelid at day 7 | |  | | | | | | |
| Dexamethasone 10 mg/kg, No | Tuncel et al. 2013 A | | | 0.00 | 0.37 | -0.72 | 0.72 | 1.00 |
| Dexamethasone 10 mg/kg, single dose | Tuncel et al. 2013 B | | | 0.00 | 0.37 | -0.72 | 0.72 | 1.00 |
|  | Tuncel et al. 2013 C | | | -1.14 | 0.39 | -1.91 | -0.37 | 0.00 |
|  | Overall effect size | | | -0.53 | 0.27 | -1.05 | 0.00 | 0.05 |
| Dexamethasone 8 mg, multiple (2 Doses) | Kargi et al. 2003 B | | | -2.13 | 0.40 | -2.90 | -1.35 | 0.00 |
| Dexamethasone 8mg, multiple (3 Doses) | Mehdizadeh et al. 2017 | | | -2.00 | 0.45 | -2.87 | -1.12 | 0.00 |
| Dexamethasone 8 mg, No | Kargi et al. 2003 A | | | -1.08 | 0.34 | -1.75 | -0.42 | 0.00 |
| Methylprednisolone 1mg/kg, No | Koc et al. 2011 A | | | -1.20 | 0.40 | -1.98 | -0.42 | 0.00 |
| Methylprednisolone 250 mg, Multiple (3 Doses) | Gurlek et al. 2009 C | | | -2.52 | 0.67 | -3.83 | -1.21 | 0.00 |
| Methylprednisolone 250 mg, No | Gurlek et al. 2009 A | | | -1.09 | 0.54 | -2.14 | -0.04 | 0.04 |
| Methylprednisolone 3mg/kg, No | Koc et al. 2011 B | | | -1.20 | 0.40 | -1.98 | -0.42 | 0.00 |
| Methylprednisolone 500 mg, Multiple (3 Doses) | Gurlek et al. 2009 D | | | -2.52 | 0.67 | -3.83 | -1.21 | 0.00 |
| Methylprednisolone 500 mg, No | Gurlek et al. 2009 B | | | -1.67 | 0.58 | -2.81 | -0.53 | 0.00 |
| Oedema Lower eyelid at day 1 |  | | | | | | | |
| Dexamethasone 10 mg/kg, No | Tuncel et al. 2013 A | | | 0.00 | 0.37 | -0.72 | 0.72 | 1.00 |
| Dexamethasone 10 mg, single dose | Hoffmann et al. 1991 | | | -0.47 | 0.29 | -1.04 | 0.10 | 0.10 |
| Dexamethasone 10 mg/kg, single dose | Tuncel et al. 2013 B | | | -1.35 | 0.40 | -2.14 | -0.56 | 0.00 |
|  | Tuncel et al. 2013 C | | | -1.35 | 0.40 | -2.14 | -0.56 | 0.00 |
|  | Overall effect size | | | -0.92 | 0.20 | -1.32 | -0.52 | 0.00 |
| Dexamethasone 8 mg, multiple (2 Doses) | Kargi et al. 2003 B | | | -1.39 | 0.35 | -2.08 | -0.70 | 0.00 |
| Dexamethasone 8mg, multiple (3 Doses) | Mehdizadeh et al. 2017 | | | -0.95 | 0.39 | -1.70 | -0.19 | 0.01 |
| Dexamethasone 8 mg, No | Kargi et al. 2003 A | | | -1.22 | 0.34 | -1.90 | -0.55 | 0.00 |
| Methylprednisolone 1mg/kg, No | Koc et al. 2011 A | | | -2.40 | 0.48 | -3.33 | -1.46 | 0.00 |
| Methylprednisolone 250mg, Multiple (3 Doses) | Gurlek et al. 2009 C | | | -1.78 | 0.59 | -2.93 | -0.62 | 0.00 |
| Methylprednisolone 250 mg, No | Gurlek et al. 2009 A | | | -1.32 | 0.55 | -2.40 | -0.24 | 0.02 |
| Methylprednisolone 3mg/kg, No | Koc et al. 2011 B | | | -2.40 | 0.48 | -3.33 | -1.46 | 0.00 |
| Methylprednisolone 500 mg, multiple (3 Doses) | Gurlek et al. 2009 D | | | -2.12 | 0.63 | -3.35 | -0.90 | 0.00 |
| Methylprednisolone 500 mg, No | Gurlek et al. 2009 B | | | -1.08 | 0.54 | -2.13 | -0.03 | 0.04 |
| Oedema Lower eyelid at day 3 |  | | | | | | | |
| Dexamethasone 8 mg, Multiple (3 Doses) | Mehdizadeh et al. 2017 | | | -1.00 | 0.39 | -1.76 | -0.24 | 0.01 |
| Methylprednisolone 1mg/kg, No | Koc et al. 2011 A | | | -1.91 | 0.44 | -2.77 | -1.05 | 0.00 |
| Methylprednisolone 250 mg, multiple (3 Doses) | Gurlek et al. 2009 C | | | -1.53 | 0.57 | -2.64 | -0.42 | 0.01 |
| Methylprednisolone 250 mg, No | Gurlek et al. 2009 A | | | -1.09 | 0.54 | -2.14 | -0.04 | 0.04 |
| Methylprednisolone 3mg/kg, No | Koc et al. 2011 B | | | -1.91 | 0.44 | -2.77 | -1.05 | 0.00 |
| Methylprednisolone 500 mg, Multiple (3 Doses) | Gurlek et al. 2009 D | | | 0.00 | 0.50 | -0.98 | 0.98 | 1.00 |
| Methylprednisolone 500 mg, No | Gurlek et al. 2009 B | | | -1.09 | 0.54 | -2.14 | -0.04 | 0.04 |
| Oedema Lower eyelid at day 5 |  | | | | | | | |
| Dexamethasone 10 mg/kg, No | Tuncel et al. 2013 A | | | 0.00 | 0.37 | -0.72 | 0.72 | 1.00 |
| Dexamethasone 10 mg/kg, single dose | Tuncel et al. 2013 B | | | -1.91 | 0.44 | -2.77 | -1.05 | 0.00 |
|  | Tuncel et al. 2013 C | | | -2.83 | 0.52 | -3.84 | -1.82 | 0.00 |
|  | Overall effect size | | | -2.30 | 0.34 | -2.95 | -1.64 | 0.00 |
| Dexamethasone 8mg, No | Kargi et al. 2003 A | | | -0.65 | 0.32 | -1.29 | -0.02 | 0.04 |
| Dexamethasone, multiple (2 Doses) | Kargi et al. 2003 B | | | -1.81 | 0.38 | -2.55 | -1.07 | 0.00 |
| Methylprednisolone 250 mg, multiple (3 Doses) | Gurlek et al. 2009 C | | | -1.89 | 0.60 | -3.06 | -0.71 | 0.00 |
| Methylprednisolone 250 mg, No | Gurlek et al. 2009 A | | | -1.33 | 0.55 | -2.42 | -0.25 | 0.02 |
| Methylprednisolone 500 mg, multiple (3 Doses) | Gurlek et al. 2009 D | | | -2.52 | 0.67 | -3.83 | -1.21 | 0.00 |
| Methylprednisolone 500 mg, No | Gurlek et al. 2009 B | | | -1.51 | 0.57 | -2.62 | -0.40 | 0.01 |
| Oedema Lower eyelid at day 7 |  | | | | | | | |
| Dexamethasone 10 mg/kg, No | Tuncel et al. 2013 A | | | 0.00 | 0.37 | -0.72 | 0.72 | 1.00 |
| Dexamethasone 10 mg, Single Dose | Hoffmann et al. 1991 | | | -0.04 | 0.29 | -0.60 | 0.52 | 0.90 |
| Dexamethasone 10 mg/kg, single dose | Tuncel et al. 2013 B | | | 0.00 | 0.37 | -0.72 | 0.72 | 1.00 |
|  | Tuncel et al. 2013 C | | | -1.14 | 0.39 | -1.91 | -0.37 | 0.00 |
|  | Overall effect size | | | -0.30 | 0.20 | -0.68 | 0.09 | 0.13 |
| Dexamethasone 8 mg, multiple (2 Doses) | Kargi et al. 2003 B | | | -1.26 | 0.35 | -1.94 | -0.58 | 0.00 |
| Dexamethasone 8 mg, multiple (3 Doses) | Mehdizadeh et al. 2017 | | | -1.18 | 0.40 | -1.96 | -0.40 | 0.00 |
| Dexamethasone 8 mg, No | Kargi et al. 2003 A | | | -0.48 | 0.32 | -1.10 | 0.15 | 0.14 |
| Methylprednisolone 1mg/kg, No | Koc et al. 2011 A | | | -1.35 | 0.40 | -2.14 | -0.56 | 0.00 |
| Methylprednisolone 250 mg, multiple (3 Doses) | Gurlek et al. 2009 C | | | -1.89 | 0.60 | -3.06 | -0.71 | 0.00 |
| Methylprednisolone 250mg, No | Gurlek et al. 2009 A | | | -1.33 | 0.55 | -2.42 | -0.25 | 0.02 |
| Methylprednisolone 3mg/kg, No | Koc et al. 2011 B | | | -1.35 | 0.40 | -2.14 | -0.56 | 0.00 |
| Methylprednisolone 500 mg, multiple (3 Doses) | Gurlek et al. 2009 D | | | -2.52 | 0.67 | -3.83 | -1.21 | 0.00 |
| Methylprednisolone 500 mg, No | Gurlek et al. 2009 B | | | -1.51 | 0.57 | -2.62 | -0.40 | 0.01 |
| Ecchymosis Lower eyelid at day 1 | | |  | | | | | |
| Dexamethasone 10 mg, No | Ozdel et al. 2006 | | | -0.37 | 0.37 | -1.09 | 0.35 | 0.31 |
| Dexamethasone 10 mg/kg, No | Tuncel et al. 2013 A | | | -1.35 | 0.40 | -2.14 | -0.56 | 0.00 |
| Dexamethasone 10 mg/kg, single dose | Tuncel et al. 2013 B | | | -1.35 | 0.40 | -2.14 | -0.56 | 0.00 |
|  | Tuncel et al. 2013 C | | | -2.70 | 0.51 | -3.69 | -1.71 | 0.00 |
|  | Overall effect size | | | -1.88 | 0.32 | -2.50 | -1.26 | 0.00 |
| Dexamethasone 8 mg, multiple (2 Doses) | Kargi et al. 2003 B | | | -2.14 | 0.40 | -2.92 | -1.36 | 0.00 |
| Dexamethasone 8 mg, multiple (3 Doses) | Mehdizadeh et al. 2017 | | | -1.08 | 0.39 | -1.85 | -0.32 | 0.01 |
| Dexamethasone 8 mg, No | Kargi et al. 2003 A | | | -1.93 | 0.38 | -2.68 | -1.18 | 0.00 |
| Methylprednisolone 1mg/kg, No | Koc et al. 2011 A | | | -3.38 | 0.57 | -4.49 | -2.26 | 0.00 |
| Methylprednisolone 250 mg, multiple (3 Doses) | Gurlek et al. 2009 C | | | -1.27 | 0.55 | -2.34 | -0.19 | 0.02 |
| Methylprednisolone 250 mg, No | Gurlek et al. 2009 A | | | 0.00 | 0.50 | -0.98 | 0.98 | 1.00 |
| Methylprednisolone 3mg/kg, No | Koc et al. 2011 B | | | -2.03 | 0.45 | -2.91 | -1.15 | 0.00 |
| Methylprednisolone 500 mg, multiple (3 Doses) | Gurlek et al. 2009 D | | | -1.41 | 0.56 | -2.51 | -0.32 | 0.01 |
| Methylprednisolone 500 mg, No | Gurlek et al. 2009 B | | | -0.57 | 0.51 | -1.57 | 0.43 | 0.27 |
| Ecchymosis Lower eyelid at day 3 | | |  | | | | | |
| Dexamethasone 8 mg, multiple (3 Doses) | Mehdizadeh et al. 2017 | | | -0.97 | 0.39 | -1.73 | -0.22 | 0.01 |
| Methylprednisolone 1mg/kg, No | Koc et al. 2011 A | | | -3.07 | 0.54 | -4.13 | -2.02 | 0.00 |
| Methylprednisolone 250 mg, Multiple (3 Doses) | Gurlek et al. 2009 C | | | -0.93 | 0.53 | -1.96 | 0.10 | 0.08 |
| Methylprednisolone 250mg, No | Gurlek et al. 2009 A | | | -0.77 | 0.52 | -1.78 | 0.25 | 0.14 |
| Methylprednisolone 3mg/kg, No | Koc et al. 2011 B | | | -2.40 | 0.48 | -3.33 | -1.46 | 0.00 |
| Methylprednisolone 500mg, multiple (3 Doses) | Gurlek et al. 2009 D | | | -2.17 | 0.63 | -3.41 | -0.94 | 0.00 |
| Methylprednisolone 500 mg, No | Gurlek et al. 2009 B | | | -1.19 | 0.54 | -2.26 | -0.13 | 0.03 |
| Ecchymosis Lower eyelid at day 5 | | |  | | | | | |
| Dexamethasone 8 mg, multiple (2 Doses) | Kargi et al. 2003 B | | | -1.45 | 0.36 | -2.15 | -0.75 | 0.00 |
| Dexamethasone 8 mg, No | Kargi et al. 2003 A | | | -0.87 | 0.33 | -1.52 | -0.22 | 0.01 |
| Dexamethasone 10 mg/kg, No | Tuncel et al. 2013 A | | | 0.00 | 0.37 | -0.72 | 0.72 | 1.00 |
| Dexamethasone 10 mg/kg, single dose | Tuncel et al. 2013 B | | | 0.00 | 0.37 | -0.72 | 0.72 | 1.00 |
|  | Tuncel et al. 2013 C | | | -0.85 | 0.38 | -1.59 | -0.10 | 0.03 |
|  | Overall effect size | | | -0.40 | 0.26 | -0.92 | 0.11 | 0.12 |
| Ecchymosis Lower eyelid at day 7 | | |  | | | | | |
| Dexamethasone 10 mg.kg, No | Tuncel et al. 2013 A | | | 0.00 | 0.37 | -0.72 | 0.72 | 1.00 |
| Dexamethasone 10 mg/kg, single dose | Tuncel et al. 2013 B | | | 0.00 | 0.37 | -0.72 | 0.72 | 1.00 |
|  | Tuncel et al. 2013 C | | | -1.35 | 0.40 | -2.14 | -0.56 | 0.00 |
|  | Overall effect size | | | -0.61 | 0.27 | -1.14 | -0.08 | 0.03 |
| Dexamethasone 8mg, Multiple (2 Doses) | Kargi et al. 2003 B | | | -1.49 | 0.36 | -2.19 | -0.79 | 0.00 |
| Dexamethasone 8 mg, multiple (3 Doses) | Mehdizadeh et al. 2017 | | | -1.68 | 0.42 | -2.51 | -0.85 | 0.00 |
| Dexamethasone 8 mg, No | Kargi et al. 2003 A | | | -0.70 | 0.33 | -1.34 | -0.06 | 0.03 |
| Methylprednisolone 1mg/kg, No | Koc et al. 2011 A | | | -1.35 | 0.40 | -2.14 | -0.56 | 0.00 |
| Methylprednisolone 250 mg, Multiple (3 Doses) | Gurlek et al. 2009 C | | | -1.89 | 0.60 | -3.06 | -0.71 | 0.00 |
| Methylprednisolone 250 mg, No | Gurlek et al. 2009 A | | | -0.77 | 0.52 | -1.79 | 0.25 | 0.14 |
| Methylprednisolone 3mg/kg, No | Koc et al. 2011 B | | | -1.35 | 0.40 | -2.14 | -0.56 | 0.00 |
| Methylprednisolone 500 mg, multiple (3 Doses) | Gurlek et al. 2009 D | | | -1.75 | 0.59 | -2.90 | -0.60 | 0.00 |
| Methylprednisolone 500 mg, No | Gurlek et al. 2009 B | | | -1.02 | 0.53 | -2.07 | 0.02 | 0.05 |

**Supplementary Figure 1 Forest plot of upper eyelid edema on day 3.**


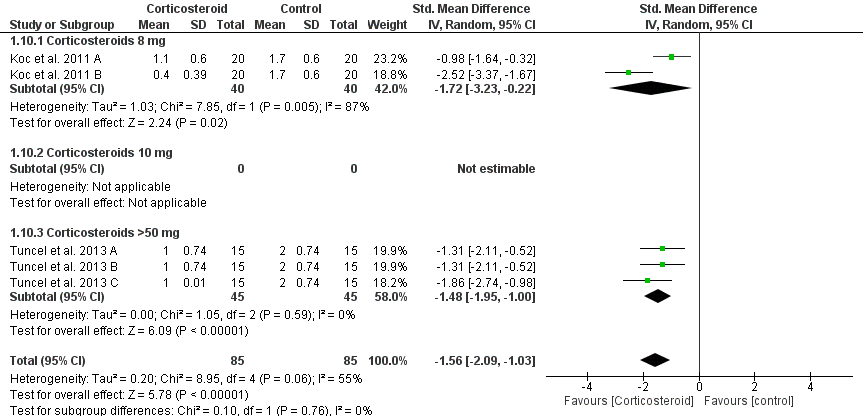


**Supplementary Figure 2 Forest plot of upper eyelid edema on day 7.**


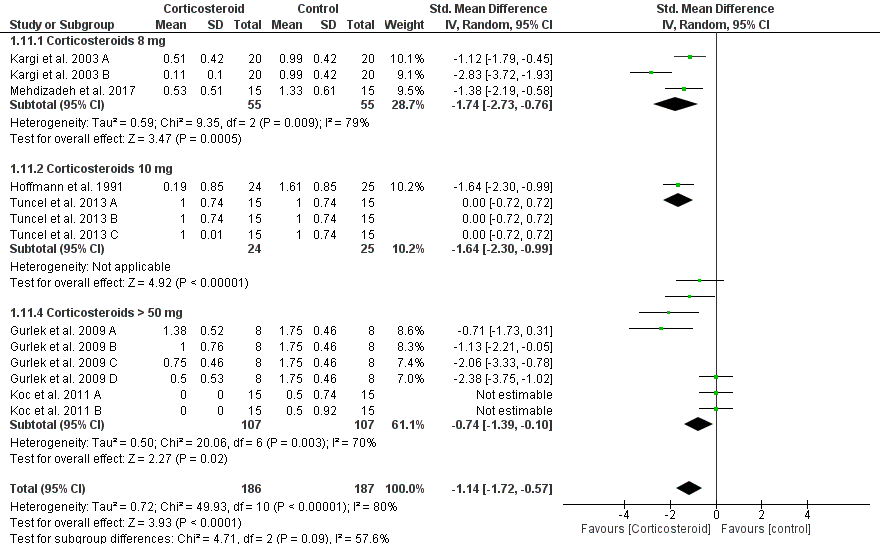


**Supplementary Figure 3 Forest plot of lower eyelid edema on day 3.**


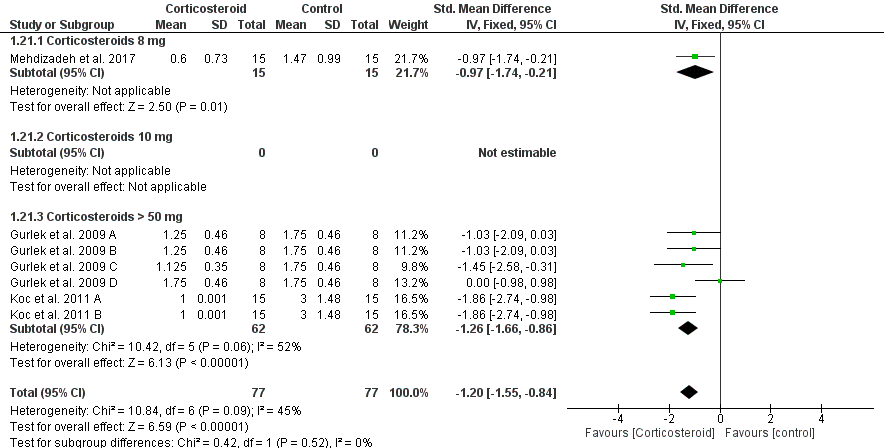


**Supplementary Figure 4 Forest plot of lower eyelid edema on day 5.**


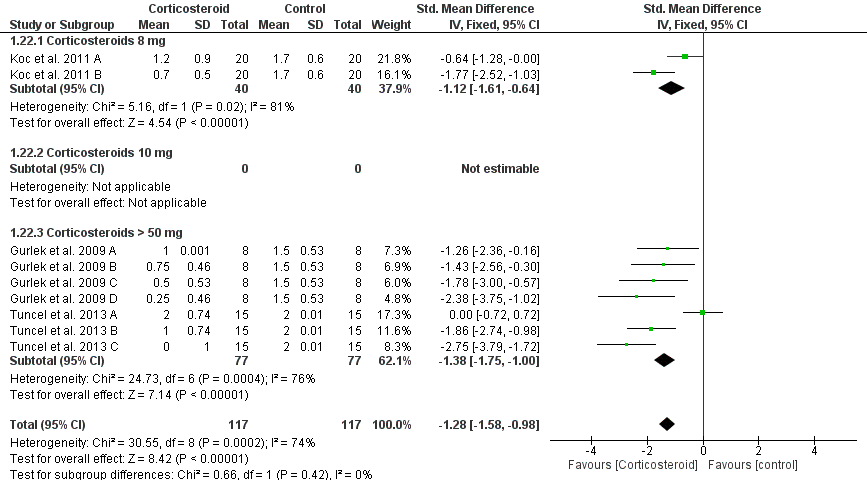


**Supplementary Figure 5 Forest plot of lower eyelid edema on day 7.**


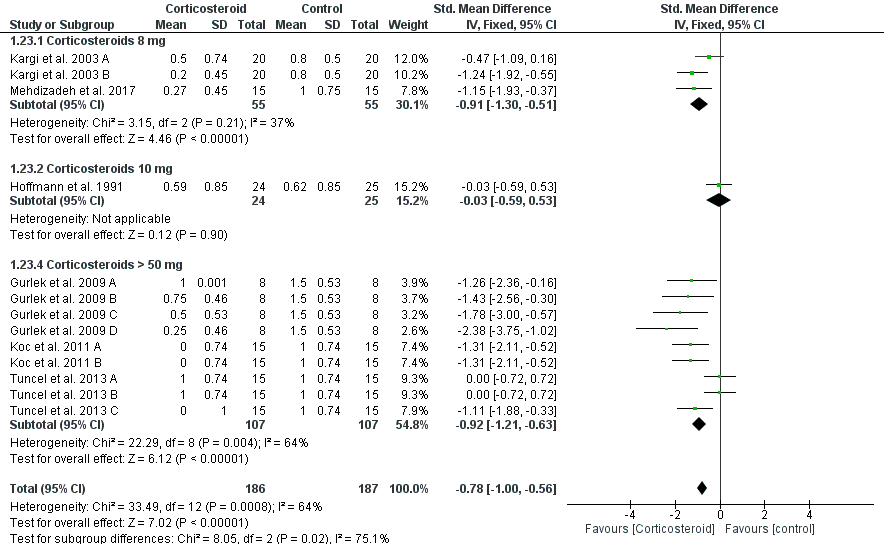


**Supplementary Figure 6 Forest plot of lower eyelid edema on day 10.**


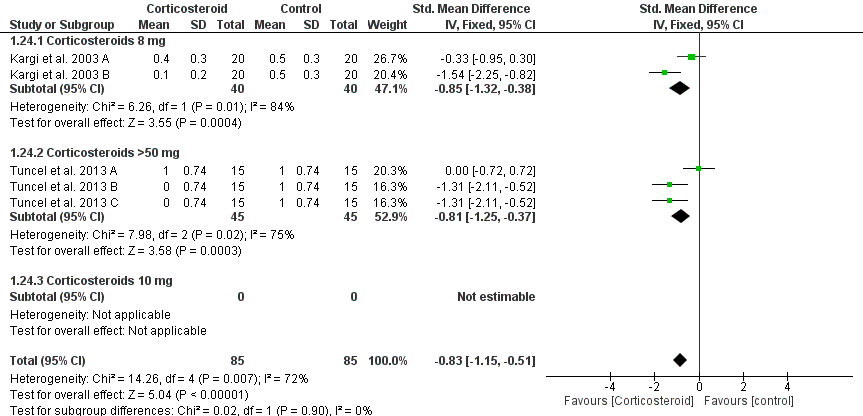


**Supplementary Figure 7 Forest plot of upper eyelid ecchymosis on day 3.**


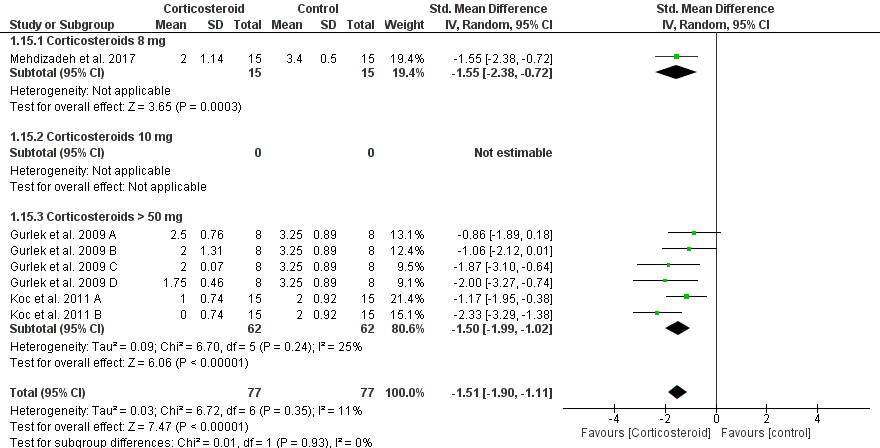


**Supplementary Figure 8 Forest plot of upper eyelid ecchymosis on day 5.**


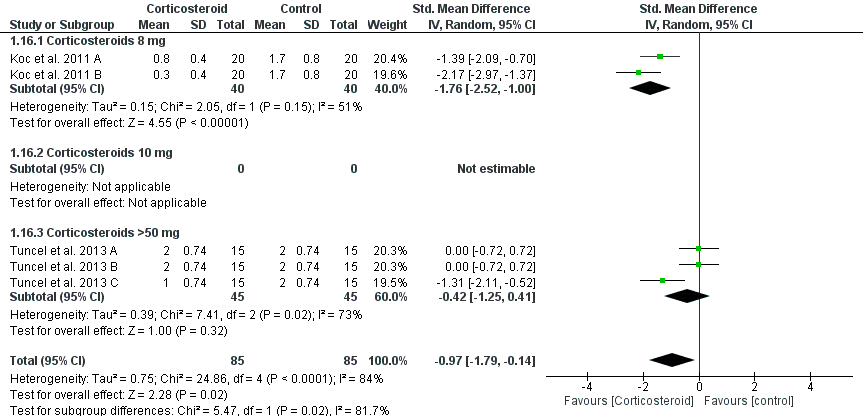


**Supplementary Figure 9 Forest plot of upper eyelid ecchymosis on day 7.**


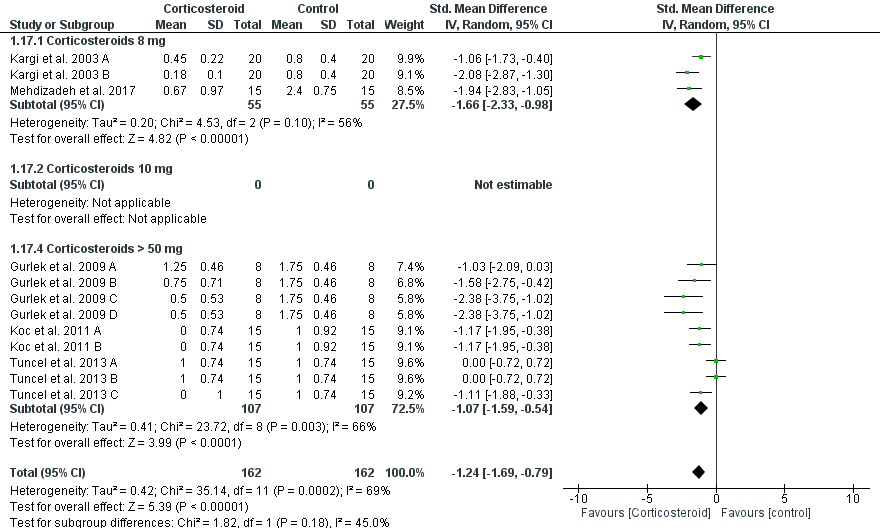


**Supplementary Figure 10 Forest plot of upper eyelid ecchymosis on day 10.**


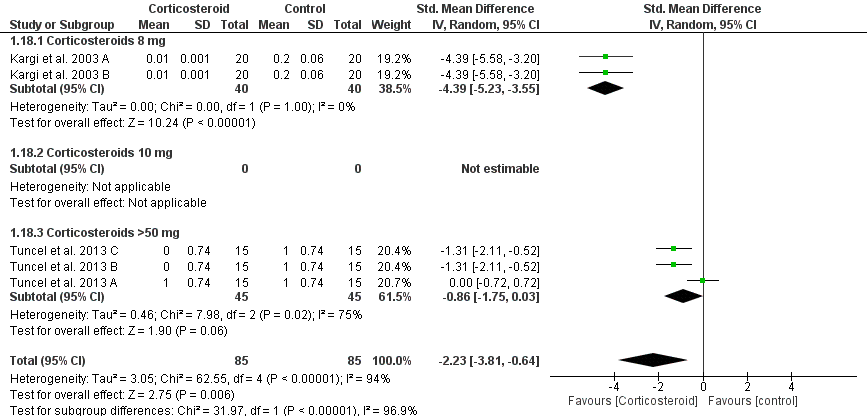


**Supplementary Figure 11 Forest plot of lower eyelid ecchymosis on day 3.**


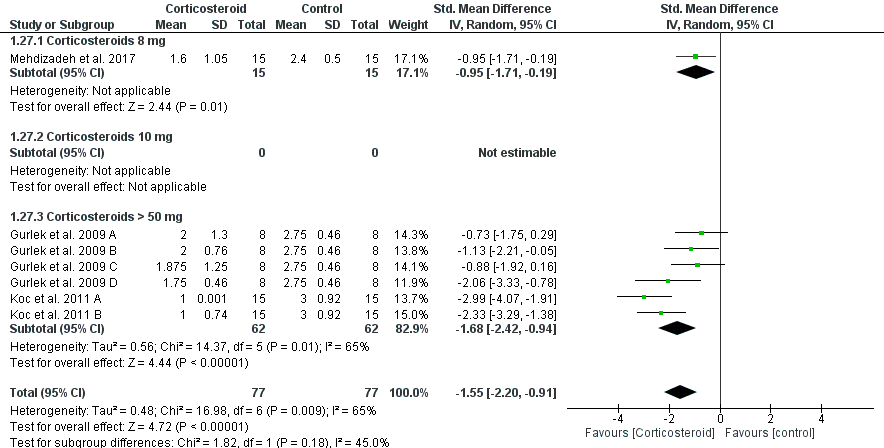


**Supplementary Figure 12 Forest plot of lower eyelid ecchymosis on day 5.**


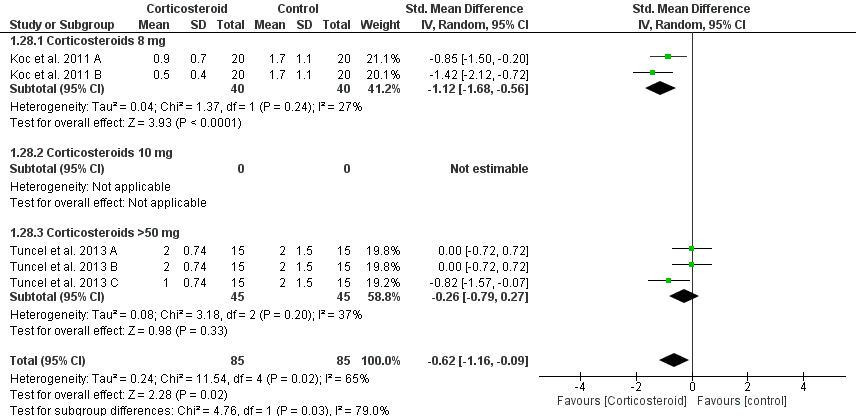


**Supplementary Figure 13 Forest plot of lower eyelid ecchymosis on day 7.**


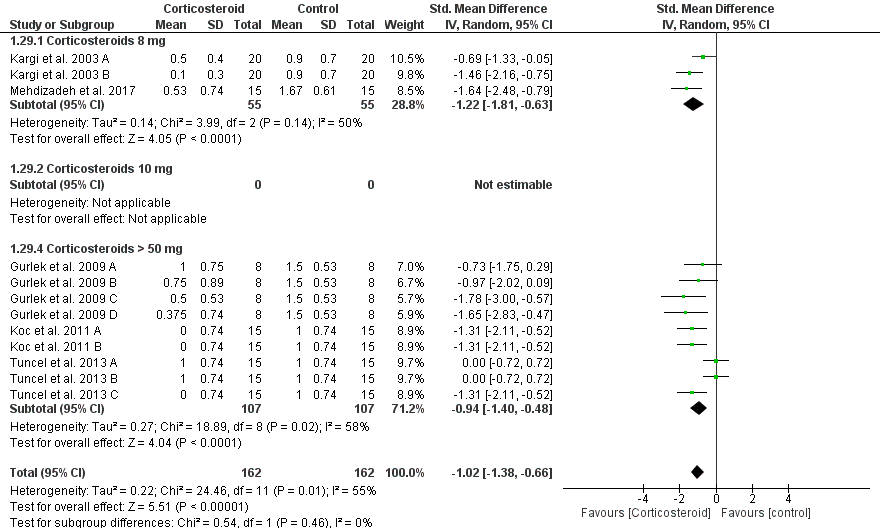


**Supplementary Figure 14 Forest plot of lower eyelid ecchymosis on day 10.**


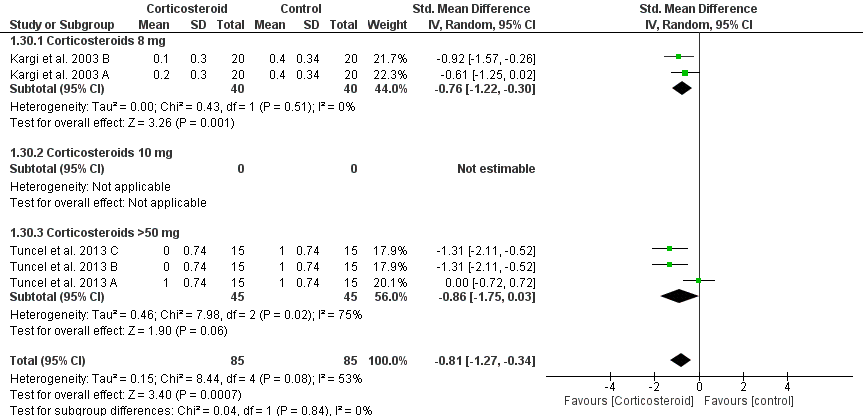


**Supplementary Figure 15 Forest plot of unspecified edema on day 3.**


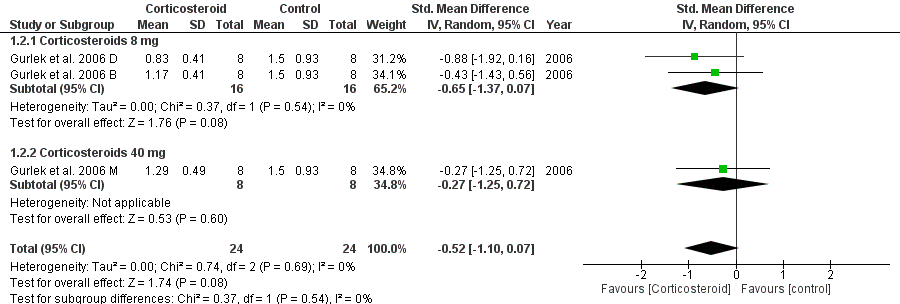


**Supplementary Figure 16 Forest plot of unspecified edema on day 7.**


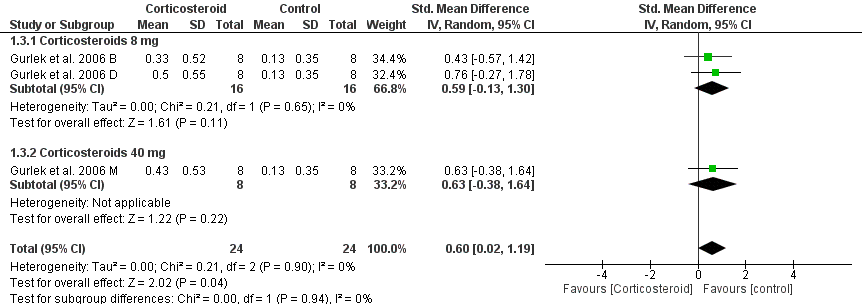


**Supplementary Figure 17 Forest plot of unspecified ecchymosis on day 3.**


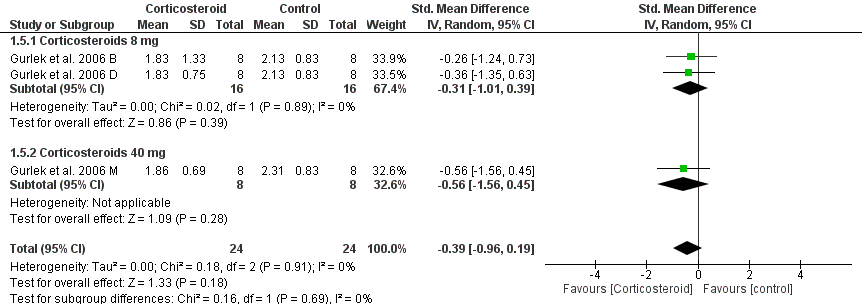


**Supplementary Figure 18 Forest plot of unspecified ecchymosis on day 7.**


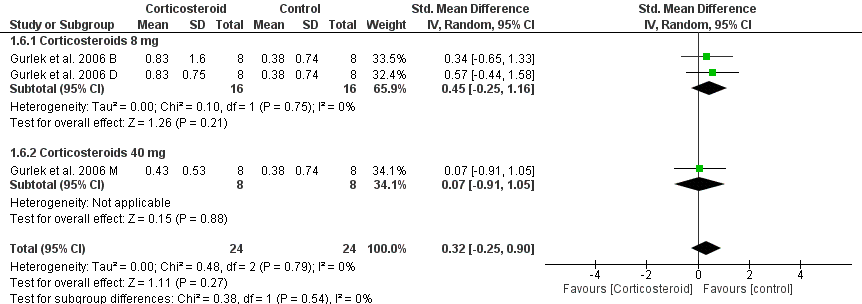

Supplement: Supplementary file 1 [file mmc1.docx]
